# TisB Protein Protects Escherichia coli Cells Suffering Massive DNA Damage from Environmental Toxic Compounds

**DOI:** 10.1128/mbio.00385-22

**Published:** 2022-04-04

**Authors:** Wei-Lin Su, Marie-Florence Bredèche, Sara Dion, Julie Dauverd, Bénédicte Condamine, Arnaud Gutierrez, Erick Denamur, Ivan Matic

**Affiliations:** a Institut Cochin, Université de Paris, INSERM U1016, CNRS UMR 8104, Paris, France; b IAME, Université de Paris, INSERM U1137, Université Sorbonne Paris Nord, Paris, France; c AP-HP, Laboratoire de Génétique Moléculaire, Hôpital Bichat, Paris, France; Max Planck Institute for Terrestrial Microbiology

**Keywords:** TisB toxin, antibiotics, colicin, *Escherichia coli*, SOS system, toxin-antitoxin

## Abstract

Toxin-antitoxin systems are genetic elements that are widespread in prokaryotes. Although molecular mode of action of many of these toxins has been identified, their biological functions are mostly unknown. We investigated the functional integration of the TisB/IstR toxin-antitoxin system in the Escherichia coli SOS genotoxic stress response network. We showed that the *tisB* gene is induced in cells exposed to high doses of the genotoxic antibiotic trimethoprim. However, we also found that TisB contributes to trimethoprim-induced lethality. This is a consequence of the TisB-induced drop in the proton motive force (PMF), which results in blocking the thymine import and therefore the functioning of the pyrimidine salvage pathway. Conversely, a TisB-induced PMF drop protects cells by preventing the import of some other toxic compounds, like the aminoglycoside antibiotic gentamicin and colicin M, in the SOS-induced cells. Colicins are cytotoxic molecules produced by *Enterobacterales* when they are exposed to strong genotoxic stresses in order to compete with other microbiota members. We indeed found that TisB contributes to E. coli*’s* fitness during mouse gut colonization. Based on the results obtained here, we propose that the primary biological role of the TisB toxin is to increase the probability of survival and maintenance in the mammalian gut of their bacterial hosts when they have to simultaneously deal with massive DNA damages and a fierce chemical warfare with other microbiota members.

## INTRODUCTION

Toxin-antitoxin (TA) systems are genetic entities composed of two genes, one coding for a toxin and another for its cognate antitoxin (1). Toxins typically inhibit essential cellular processes and components, such as translation, DNA replication, membranes, and cytoskeleton. TA systems are classified in different types according to their genetic structure and the mode of toxin neutralization. TA systems are widely distributed on plasmids, bacteriophages, and bacterial chromosomes. While the molecular mode of action of many TA systems has been extensively studied, their biological roles are still mostly unknown (1). Identification of TA systems’ mode of action responds to the mechanistic “how” but not necessarily to the evolutionary/ecological “why” question. Notable exceptions are some plasmid-borne TA systems, which ensure plasmid stabilization, such as the F plasmid-borne CcdA/CcdB TA system (2). Identification of TA systems’ biological functions is impeded by the fact that the inactivation of genes coding for most of the TA systems has no phenotype under laboratory conditions (2). For many toxins, phenotypes were observed only when they were produced over physiological levels (2), which is often artifact prone.

Important information about TA systems’ biological roles may come from the understanding of how the control of their expression is integrated in cellular signaling pathways. Particularly convenient for this purpose is when regulatory mechanisms are well characterized. One such example is the SOS regulon in Escherichia coli, which is composed of about 50 genes, including six TA systems (3). In unstressed cells, the SOS regulon genes are repressed by the LexA protein. The LexA-controlled SOS regulon has an ancient origin, and is present in a large number of widely diverged bacterial phyla (4). This regulon can be induced by a wide variety of stressors, all of which cause the accumulation of persistent single-strand DNA (ssDNA). The RecA protein polymerizes on the ssDNA, which activates its coprotease activity, which in turn promotes the proteolytic autocleavage of the LexA protein, thus activating the transcription of the SOS regulon genes. When the DNA damage is repaired, persistent ssDNA disappears and the SOS regulon is repressed again. Because the SOS regulon was the first identified as a response to genotoxic stress, and because many LexA-regulated genes code for DNA damage-processing functions, roles of other genes that are not obviously involved in the DNA repair were largely ignored. This is the case for six LexA-regulated chromosomal TA systems: DinQ/AgrB, HokE/SokE, SymE/SymR, TisB/IstR, YafQ/DinJ, and YafN/YafO (3). An unanswered question is why these TA systems are integrated in such a complex, strictly regulated genotoxic stress response.

The best-studied LexA-regulated TA system is TisB/IstR (5) (Fig. S1A). This type I TA system is composed of the TisB protein toxin and the IstR RNA antitoxin. The *tisB* gene is repressed by LexA, while *istR* is constitutively expressed (5). In unstressed cells, the toxin is not synthetized because the 5′ untranslated region (UTR) secondary structure of *tisB* primary transcript prevents its translation. Processing of the *tisB* primary transcript generates translationally active mRNA, but the binding of the antitoxin *istR* RNA triggers RNase III cleavage and *tisB* mRNA degradation (5). Under SOS regulon-inducing conditions, massively synthetized *tisB* mRNA out-titrates *istR* RNA and the TisB protein is produced. TisB is a small hydrophobic protein that targets the cytoplasmic membrane and disrupts the proton motive force (PMF) (6), which is necessary for many membrane transactions, including nutrient uptake and ATP production (Fig. S1A). While the overproduction of the plasmid-borne *tisB* gene, with or without concomitant SOS induction, provokes a severe decrease in transcription, translation, and DNA replication and results in rapid cell death, the induction of the chromosomal *tisB* gene by SOS-inducing treatments does not kill the cells (5).

10.1128/mbio.00385-22.1 FIG S1 Regulation of the *tisB* gene expression, folate cycle, and dTTP synthesis. Download FIG S1, PDF file, 0.6 MB. Copyright © 2022 Su et al. 2022 Su et al. https://creativecommons.org/licenses/by/4.0/ This content is distributed under the terms of the Creative Commons Attribution 4.0 International license.

It was hypothesized that upon SOS induction, TisB renders cells transiently metabolically inactive and consequently tolerant to antibiotics (7). However, there is no unambiguous evidence for the involvement of TisB in the drug-induced persistence to antibiotics. For example, it was reported that ciprofloxacin (CIP), a fluoroquinolone antibiotic that poisons DNA gyrase, induces an SOS response and increases TisB-dependent persistence to CIP (7). However, another study reported that TisB-dependent increased persistence to CIP is due not to SOS regulon induction by CIP but to the presence of spontaneously induced persisters that were present in the bacterial population prior to CIP treatment (8). It was also shown that TisB does not impact persistence of growing and stationary-phase E. coli cells to another SOS-inducing fluoroquinolone antibiotic, ofloxacin (9, 10). These data indicate that drug-induced TisB production does not prevent generation of DNA damages by fluoroquinolones and that it does not contribute to the repair of these damages. So the question of why TisB/IstR toxin-antitoxin system is integrated in the SOS genotoxic stress response network remains open.

In order to answer this question, we investigated the impact of TisB on the survival of E. coli cells treated with the antibiotic trimethoprim (TMP). TMP is a competitive inhibitor of bacterial dihydrofolate reductase, which prevents the synthesis of methionine, glycine, purines and dTTP (11) (Fig. S1B). In rich growth medium, TMP-bactericidal effect resides entirely on the intracellular dTTP depletion, which affects DNA synthesis, leads to DNA damage, SOS induction, and cell death. The advantage of using TMP instead of fluoroquinolones is that the bactericidal effect of TMP can be reversed by the addition of thymine or thymidine, which can be used for dTTP synthesis via a pyrimidine salvage pathway (12) (Fig. S1C). Importantly, the import of thymine or thymidine is PMF dependent, which is affected by TisB. We also tested if TMP-induced TisB impacts the PMF-dependent import of some other compounds: lactose, gentamicin (GM), and colicin M (ColM). Finally, we investigated if TisB may play a role in the colonization of the mouse intestine by E. coli. Based on the results obtained, we propose that the primary role of TisB is to transiently block the import of host and/or microbiota-produced toxic compounds into cells suffering massive DNA damage and thus to increase the probability of survival and maintenance in the mammalian gut.

## RESULTS

### Impact of TisB on SOS induction and the survival of TMP-treated cells.

For this study, we used E. coli K-12 MG1655 strain, referred to here as wild type (WT), and its Δ*tisB* derivative (Table S1). To investigate which of the TisB-dependent phenotypic alterations contribute to the survival of stressed cells, we treated cultures of WT and Δ*tisB* strains growing in LB medium. We first tested the susceptibility of WT cells from different growth phases to different TMP concentrations (Fig. S2A and B) and decided that the treatment of exponentially growing cultures having around 2 × 10^8^ CFU/mL (optical density at 600 nm [OD_600_] = 0.6) and a TMP concentration of 10 μg/mL, i.e., 20 × MIC (Table S2), were most suitable for our study. This concentration of TMP was chosen because further increase of the TMP concentration did not further decrease survival, i.e., the dose-response relationship displayed the Eagle effect (13, 14). This cell concentration was chosen because cells at this growth phase were more susceptible to TMP. The stationary-phase cells (OD_600_ = 2) were barely impacted.

10.1128/mbio.00385-22.2 FIG S2 TMP killing activity depends on the cell culture growth phase, thymidine, the pyrimidine salvage pathway, and DNA replication activity. Download FIG S2, PDF file, 0.3 MB. Copyright © 2022 Su et al. 2022 Su et al. https://creativecommons.org/licenses/by/4.0/ This content is distributed under the terms of the Creative Commons Attribution 4.0 International license.

10.1128/mbio.00385-22.6 TABLE S1 Bacterial strains and plasmids used in this study. Download Table S1, PDF file, 1.5 MB. Copyright © 2022 Su et al. 2022 Su et al. https://creativecommons.org/licenses/by/4.0/ This content is distributed under the terms of the Creative Commons Attribution 4.0 International license.

10.1128/mbio.00385-22.7 TABLE S2 MICs for the studied strains of different antibiotics. Download Table S2, PDF file, 0.9 MB. Copyright © 2022 Su et al. 2022 Su et al. https://creativecommons.org/licenses/by/4.0/ This content is distributed under the terms of the Creative Commons Attribution 4.0 International license.

We first confirmed that TMP causes dTTP depletion, which blocks DNA replication and induces an SOS response. This conclusion is based on the observation that addition of thymidine, which can be used for dTTP synthesis *via* a pyrimidine salvage pathway, completely abolished the TMP cytotoxic effect (Fig. S1BC and C). We further validated this conclusion by modulating the flow of thymine and thymidine through the pyrimidine salvage pathway (Fig. S2C), by deleting *deoC* and *deoR*. The deletion of *deoC*, which results in decreased utilization of thymine and thymidine for energy production, thus allowing cells to use it for nucleotide synthesis, increased survival. Deletion of *deoR*, which results in an increased thymine catabolism by favoring its flux toward the tricarboxylic acid (TCA) cycle, decreased survival. Finally, we showed that delaying dTTP depletion, by introducing the *dnaA*(Sx) allele, which reduces DNA replication initiation frequency, increased survival 20-fold (Fig. S2D).

**FIG 1:**
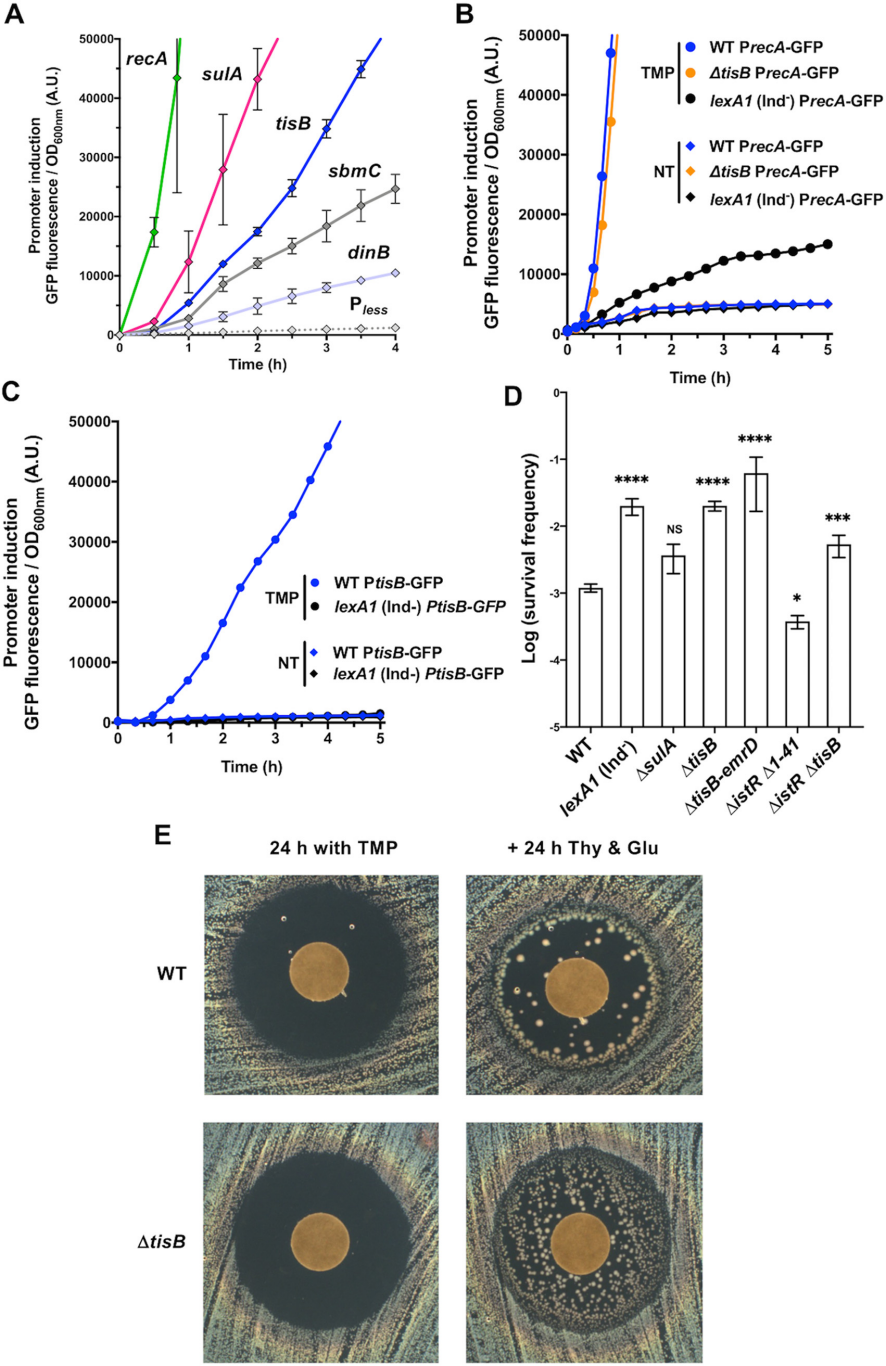
Induction and involvement of different SOS genes in the survival of cells treated with TMP. (A) Kinetics of the induction of five SOS genes by 20 × MIC of TMP measured by following the GFP fluorescence intensity. The promoterless (P_less_) plasmid was used as a negative control. Each dot represents the mean value (with standard error of the mean [SEM]) of results obtained from 3 independent experiments. (B) The induction of the SOS response in cells treated with 20 × MIC of TMP measured using a P*recA*-GFP reporter. (C) The induction of *tisB* in cells treated with 20 × MIC of TMP measured using a P*tisB*-GFP reporter. (B and C) A *lexA1* (Ind^−^) strain and no-treatment condition (NT) were used as negative controls for SOS induction. The results are representative of 3 independent experiments. (D) Survival of mutants of several SOS genes of 20 h of 20 × MIC of TMP treatment. Each bar represents the mean (with SEM) of results obtained from at least 4 independent experiments. The Mann-Whitney test was used for comparison with the WT unless otherwise indicated. *, *P* < 0.05; ***, *P* < 0.001; ****, *P* < 0.0001; NS, *P* > 0.05. (E) TDtest. (Left) Susceptibility to TMP, which was spotted on the discs, after 24 h of incubation. (Right) Colonies of surviving bacteria within the growth inhibition zone visualized after an additional 24 h of incubation with glucose (Glu), which provides energy for growth, and thymidine (Thy), which counteracts the effects of TMP, which were spotted on the discs.

We investigated the induction of the *tisB* gene by TMP treatment in our experimental conditions. We also compared the kinetics of *tisB* induction with the induction of other LexA-controlled genes. We used a collection of low-copy-number plasmids, each carrying a promoter region of a SOS gene inserted upstream of a gene encoding a fast-folding GFPmut2 (Fig. 1A; see Fig. S3A for all 12 promoters). P*recA* was the first to be induced after ∼20 min, followed by P*lexA*, P*sulA*, and P*recN*, while P*tisB* was induced after ∼1 h of treatment. Other promoters were induced later and at a much lower rate. We performed the same experiment using CIP as an SOS-inducing agent and found that the pattern of induction of the tested SOS genes was very similar to the one obtained with TMP (Fig. S3B). Finally, we showed that the induction of the P*recA*-GFP and P*tisB*-GFP reporters by TMP treatment is due to a *bona fide* SOS induction regulation; i.e., it is LexA dependent (Fig. 1B and C). Importantly, although P*recA*, P*lexA*, and P*ruvA* were induced in cells treated with 0.1 × MIC of TMP, P*tisB* was not (Fig. S3D).

10.1128/mbio.00385-22.3 FIG S3 Kinetics of the induction of the SOS genes by antibiotic treatments. Download FIG S3, PDF file, 0.2 MB. Copyright © 2022 Su et al. 2022 Su et al. https://creativecommons.org/licenses/by/4.0/ This content is distributed under the terms of the Creative Commons Attribution 4.0 International license.

Next, we investigated the involvement of the SOS regulon induction in the modulation of the capacity of cells to survive 20 h of treatment with 20 × MIC of TMP. We tested the survival of a *lexA1* (Ind^−^) mutant, which cannot induce the SOS regulon, and of a *ΔsulA* mutant, which cannot prevent cell division, i.e., cells do not filament (Fig. 1D). These two mutants were chosen because we previously found that *lexA1* (Ind^−^) and Δ*sulA* mutants have, respectively, decreased and increased susceptibility to sub-MIC of TMP (15), which correlates with the MICs of these strains (Table S2). The survival of *lexA1* (Ind^−^) of the lethal TMP treatment was 10-fold higher than the survival of the WT strain, suggesting that the induction of one or more of the SOS functions contribute to the death of TMP-treated cells. Inactivation of the *sulA* gene had no significant impact on the survival of TMP-treated cells. However, the survival of the *ΔtisB* mutant was 20-fold higher than that of the WT strain (Fig. 1D), although the MICs for these two strains are not different (Fig. 1E and Table S2). Such a discrepancy between MIC and survival of lethal antibiotic treatment is a hallmark of tolerance to antibiotics (16). Importantly, by using the P*recA*-GFP reporter, we showed that the deletion of the *tisB* gene did not affect the induction of the SOS regulon, as indicated by the induction of the *recA* gene promoter (Fig. 1B), which could impact cell DNA repair capacity and survival of TMP treatment.

Because the *tisB* gene is upstream of the *emrD* gene, which codes for a multidrug efflux pump (Fig. S1A), we verified if the deletion of the *tisB* gene has a polar effect on the *emrD* gene and consequently increases cell capacity to deal with TMP (Fig. 1D). However, this was not the case, because *ΔtisB* and *ΔtisB-emrD* mutants had the same susceptibility. TisB’s antitoxin IstR alone has no impact on TMP cytotoxicity, because the survival of the *ΔistR ΔtisB* strain was not significantly different from that of the *istR^+^ ΔtisB* strain (Fig. 1D). Higher production of the TisB protein in the *ΔistR Δ1–41* strain (5), which lacks both IstR and the small DNA region coding for the 5′ UTR structure in *tisB* mRNA that delays TisB translation, resulted in reduced survival compared to the WT strain (Fig. 1D). This result confirms that strictly regulated transcription of *tisB* gene is required for appropriate TisB functioning.

We also used a pBAD plasmid carrying the *tisB* gene under the control of an arabinose-inducible promoter to evaluate the impact of the modulation of TisB production on cell viability. Strong *tisB* overexpression upon induction by l-arabinose blocks E. coli’s growth even in the absence of any treatment (17). Therefore, we did not add the inducer, and we observed that the leakiness of the promoter ensures a TisB production sufficient to reduce the survival of the Δ*tisB* mutant treated with lethal TMP dose, while it did not have a significant impact on the survival of the WT strain (Fig. S2E). This result confirms that the absence of the TisB protein alone, and not a secondary consequence of the *tisB* gene deletion, is responsible for most, if not all, of the killing resulting from the SOS regulon induction with TMP.

### Impact of TisB on the morphology of TMP-treated cells.

**FIG 2:**
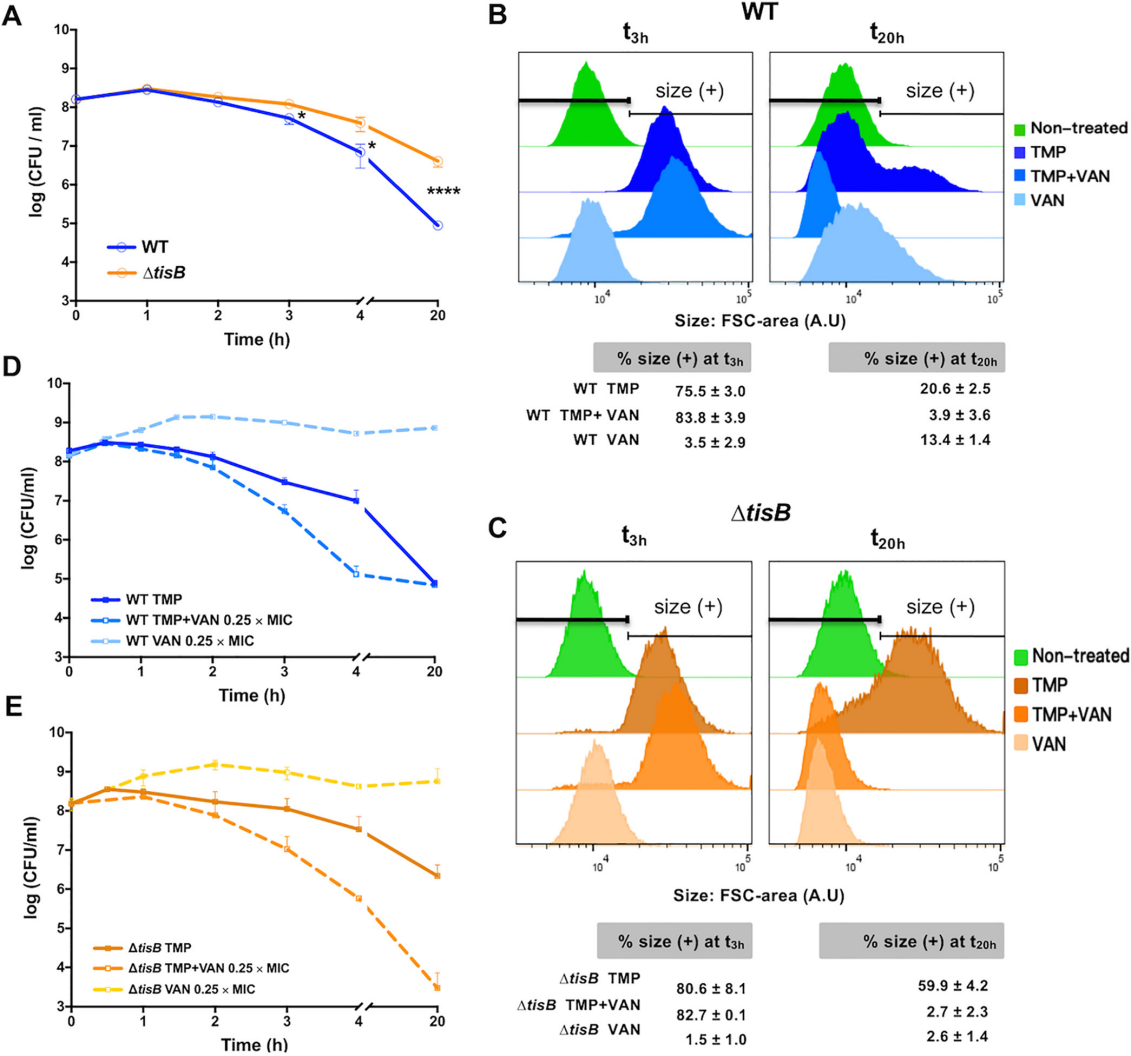
Morphology and survival of antibiotic-treated cells. WT and Δ*tisB* cells were treated with (A) 20 × MIC of TMP or (D and E) 0.25 × MIC of vancomycin (VAN) or the combination of TMP and VAN, and the killing kinetics are presented as survival over time. Each dot represents the mean value (with SEM) of results obtained from 3 independent experiments (Mann-Whitney test; *, *P* < 0.05; ****, *P* < 0.0001). (B and C) The WT (B) and Δ*tisB* (C) cell size (forward scatter [FSC] area) distribution after 3 h and 20 h of antibiotic treatment determined by flow cytometry (data from panels D and E, respectively). Fifty thousand cells were analyzed for each condition. Stationary-phase untreated cells were used as a control for cell size. The histograms are representative of 3 independent experiments. The tables show the percentage of cells with a size higher than that of the control population of stationary-phase untreated cells. The values are means (with SEM) from 3 independent experiments.

The comparison of the kinetics of TMP-induced killing of the WT and Δ*tisB* mutant strains, measured by CFU counts, showed that their survival was not significantly impacted during the first 3 h of treatment (Fig. 2A). After 3 h, the survival of both strains started decreasing, albeit at different rates; i.e., CFU of the WT were reduced faster than CFU of the Δ*tisB* mutant. After 4 h of treatment, the fraction of surviving WT and Δ*tisB* mutant cells decreased 22- and 4-fold, respectively. After 20 h of treatment, the fractions of surviving WT and Δ*tisB* mutant cells decreased 1,780- and 37-fold, respectively.

We compared the sizes of the WT and Δ*tisB* cells during TMP treatment. After 3 h of treatment, the WT and Δ*tisB* cells had similar size distributions (Fig. 2B and C). However, the two strains displayed different cell size distributions after 20 h of treatment, i.e., Δ*tisB* cells were longer than WT cells. Because dormant stationary-phase cells are usually smaller than growing cells, it is unclear if surviving *ΔtisB* cells are dormant or metabolically active growing cells. In order to address this question, we took advantage of the observation that the combination of TMP and vancomycin (VAN) displays a synergistic effect on E. coli (18). VAN inhibits peptidoglycan synthesis but is ineffective on E. coli because it cannot penetrate the outer membrane of Gram-negative bacteria due to its large size. However, VAN can affect Gram-negative bacteria when their outer membrane is damaged. The fact that TMP and VAN have a synergistic effect suggests that TMP treatment damages the outer membrane, thus allowing VAN to interact with its target. Cells that are not affected by TMP, like nongrowing stationary-phase cells, should not be affected by VAN either. In addition, peptidoglycan synthesis is active in growing cells and slows down in stationary-phase cells (19).

Therefore, we used VAN as a probe to investigate if peptidoglycan synthesis is active in TMP-treated cells. WT and *ΔtisB* cells were treated with TMP and/or with 0.25 × MIC of VAN (Table S2) during 20 h. We showed that the TMP treatment sensitized WT cells to VAN, which accelerated the killing of the cells. However, the fraction of the WT cells surviving 20 h of TMP+VAN treatment was the same as with TMP treatment alone (Fig. 2D). The WT cells treated with TMP+VAN were also significantly smaller than cells treated with TMP alone (Fig. 2B). These cells had the same size as stationary-phase cells, which further confirmed that WT survivors are small nongrowing cells. However, the treatment of *ΔtisB* with TMP+VAN accelerated the killing and diminished 725-fold the fraction of cells surviving 20 h of treatment compared to that of TMP treatment alone, to a level 23-fold lower than that of the WT cells (Fig. 2E). In addition, the *ΔtisB* cells were smaller after TMP+VAN than after TMP treatment alone (Fig. 2C), which indicates that most of the *ΔtisB* cells surviving TMP treatment are bigger, metabolically active cells.

### Impact of TisB on the membrane potential and ATP pool in TMP-treated cells.

**FIG 3:**
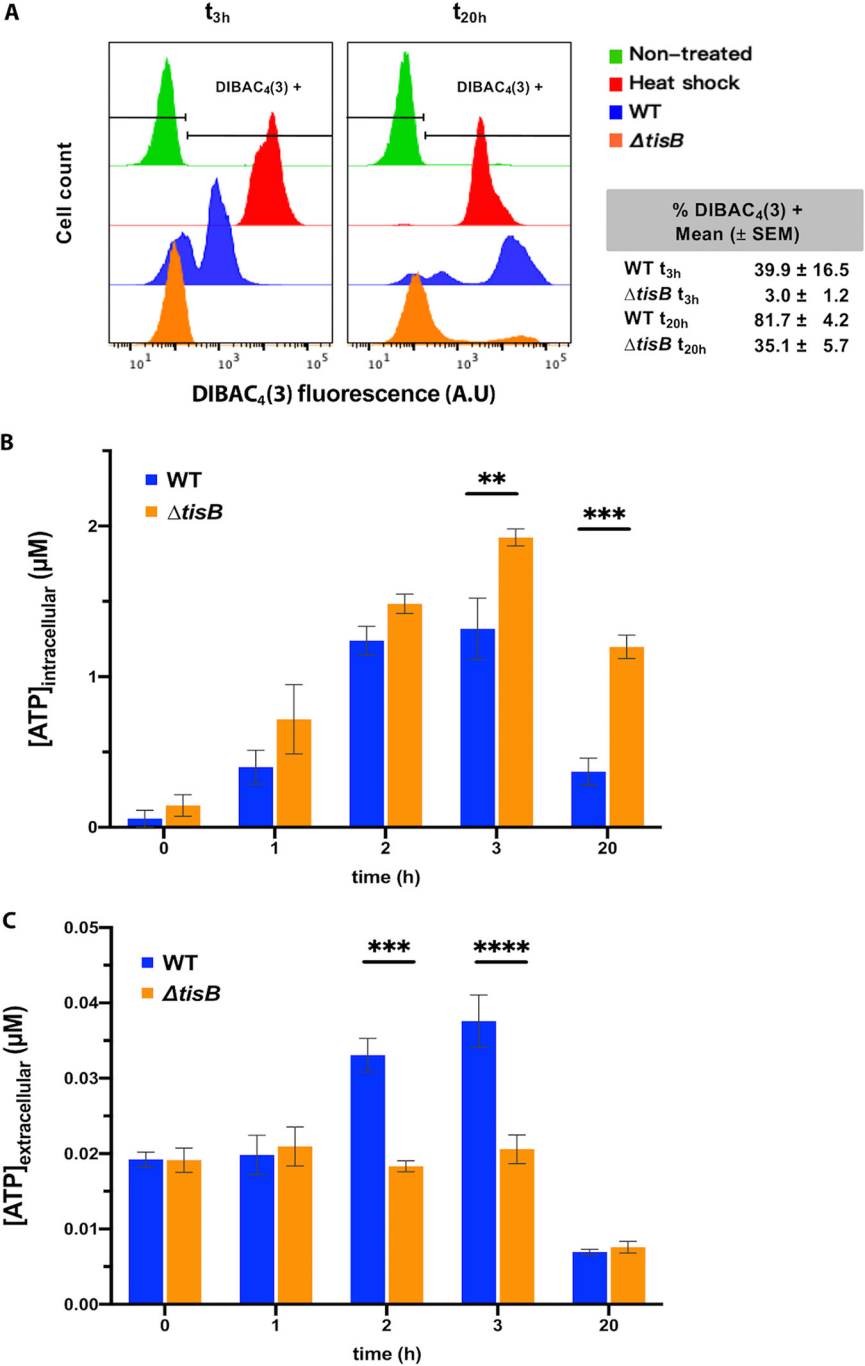
Membrane permeability and ATP pool in cells treated with 20 × MIC of TMP. (A) The cell permeability assayed by DIBAC_4_(3) staining after 3 h and 20 h of TMP treatment is determined by flow cytometry. Each histogram represents the distribution for 50,000 cells analyzed. Stationary-phase untreated cells were used as a negative control. Heat-killed cells were used as a positive control. The presented results are representative of at least 3 independent experiments. The tables show the percentage of cells with a size higher than that of the control population of stationary-phase untreated cells. The values are means (with SEM) from 3 independent experiments. (B and C) Intracellular (B) and extracellular (C) ATP concentrations during TMP treatment. The results are the means (with SEM) of results obtained from 3 independent experiments (Student's *t* test; **, *P* < 0.01; ***, *P* < 0.001; ****, *P* < 0.0001).

It was reported that TisB overproduction affects cytoplasmic membrane integrity and disrupts the PMF (6), which is necessary for many membrane transactions, including ATP production (17) (Fig. S1A). Because TisB-dependent decrease of ATP levels was proposed to induce persistence to CIP (7), we examined how the TMP treatment impacts cell membrane and ATP level. First, we tested membrane potential using bis-(1,3-dibutylbarbituric acid)-trimethine oxonol [DIBAC_4_(3)], which enters cells with depolarized membranes and flow cytometry (20). We found that the TMP-treated WT cells started to be stained after 3 h of treatment, whereas the *ΔtisB* strain was not stained at this time point (Fig. 3A). After 20 h of TMP treatment, 80% of WT and 35% of *ΔtisB* cells were stained with DIBAC_4_(3).

Second, we investigated the impact of TMP treatment on the ATP pool in WT and *ΔtisB* cells and in the growth medium. We found that the intracellular ATP amount increased in both strains, which corroborates reports showing that treatments with UV radiation, bleomycin, and DNA gyrase inhibitors increase intracellular ATP concentration in E. coli (21, 22). After 2 h of TMP treatment, the amount of ATP stopped increasing in WT but continued to increase in the *ΔtisB* mutant. After 20 h of treatment, ATP amount was still higher in *ΔtisB* than in WT cells (Fig. 3B). The observed difference between the two strains is probably due to the TisB-mediated drop in ATP production in WT cells. Measurement of the extracellular ATP amounts showed that they stayed constant during the first 3 h in the medium with *ΔtisB* cells but that they increased in the medium with WT cells (Fig. 3C). This increase of the ATP in the medium with treated WT cells most probably results from leakage from dead cells and/or cell lysis.

Therefore, because WT cells have less ATP and reduced survival compared to *ΔtisB* cells, it can be concluded that, unlike what was proposed for the persisters to CIP, the intracellular ATP levels were not anticorrelated with the survival of TMP treatment.

### Impact of TisB on death of cells after TMP treatment.

The survival measured by CFU counts is the sum of cells’ ability to survive during exposure to a drug and their abilty to recover and start growing after drug removal. This may explain why the frequency of the DIBAC_4_(3)-stained cells detected by flow cytometry after 20 h of TMP treatment did not correspond to the CFU-based survival frequency (Fig. 1D and 3A). However, it should be noted that DIBAC_4_(3) stains dead cells (20) but that it also stains live cells with depolarized membranes. For this reason, we decided to investigate the kinetics of the post-TMP treatment staining with DIBAC_4_(3) but also with Alexa Fluor 633 hydrazide (AFH633), which detects carbonylated macromolecules and is a reliable marker of cell death (20, 23). As a positive control, we showed that both dyes stain heat-killed cells.

**FIG 4:**
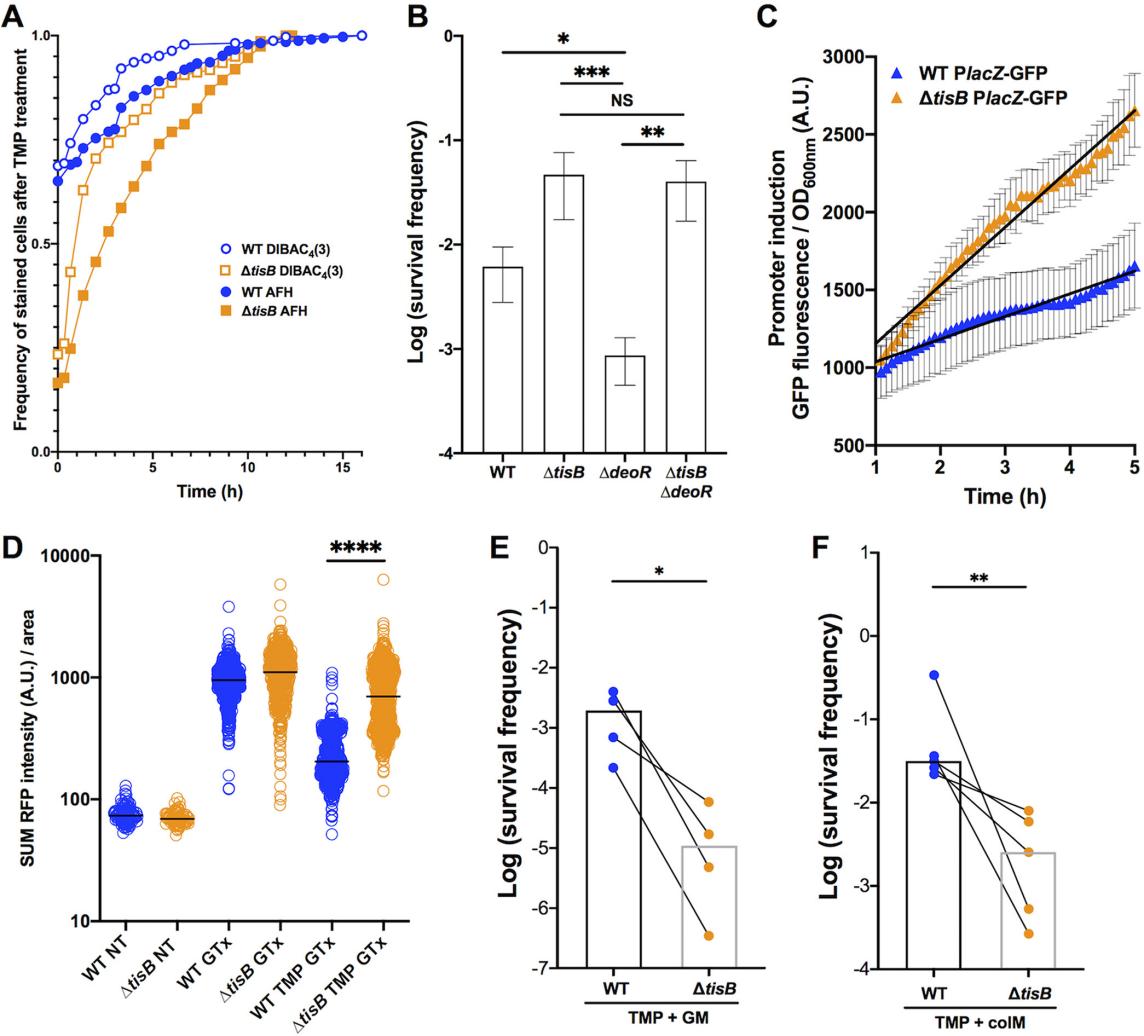
Impact of TisB on cell death kinetics after TMP treatment and on the import of different extracellular compounds during TMP treatment. (A) The kinetics of AFH633 and DIBAC_4_(3) cell staining after treatment with 20 × MIC of TMP is plotted as the frequency of stained cells over time. For this experiment, cells were loaded into the channels of a “mother machine” microfluidic device, incubated for 16 h in LB medium supplemented with DIBAC_4_(3) and AFH633, and monitored with a fluorescence microscope. The results are from 4 independent experiments; a total of 329 and 657 WT and Δ*tisB* cells, respectively, were analyzed. (B) The impact of TisB on the pyrimidine salvage pathway was measured by the survival of the WT, Δ*tisB*, Δ*deoR*, and Δ*tisB* Δ*deoR* strains treated with 20× MIC of TMP. Each bar represents the mean (± SEM) of data obtained from at least 3 independent experiments (Mann-Whitney test; *, *P* < 0.05; **, *P* < 0.01; ***, *P* < 0.001; NS, *P* > 0.05). (C) The import of lactose was measured using the P*lacZ*-GFP reporter. Lactose was added after 1 h of treatment with 20× MIC of TMP. The obtained values were corrected by subtracting values from the control condition without lactose. Each dot represents the mean (with SEM) of 5 independent experiments. The lines represent the linear regression (slope mean value [SEM]: WT = 146 [4.2], Δ*tisB *= 374.7 [7.9]; *r*^2^: WT = 0.9619, Δ*tisB *= 0.9791). The Prism linear regression slope comparison test was used. ****, *P*, < 0.0001. (D) The import of the gentamicin-Texas Red (GTx) conjugate in WT and Δ*tisB* cells monitored using fluorescence microscope. Each dot represents the value of summed fluorescence corrected by the area for a single cell. The results shown are obtained from 3 independent experiments, and a total of 112, 85, 529, 430, 517 and 597 cells were analyzed for the WT NT, Δ*tisB* NT, WT GTx, Δ*tisB* GTx, WT TMP GTx, and Δ*tisB* TMP GTx conditions, respectively. For this experiment, TMP and GTx were used at 20 × MIC and MIC, respectively. Mann-Whitney test, ****p-value <  0.0001. (E) Gentamicin (GM) at the MIC was added to the cultures after 3 h of treatment with 20 × MIC of TMP, and survival after 20 h was determined by CFU counts. (F) Colicin M (ColM) at the MIC was added to the cultures after 3 h of 20 × MIC of TMP treatment, and survival after 20 h was determined by CFU counts. (E and F) Paired results from 4 and 5 independent experiments are presented (Mann-Whitney test; *, *P* < 0.05; **, *P* < 0.01). Each bar represents the median of the data.

We loaded the cells, immediately after the removal of TMP, into the channels of a “mother machine” microfluidic device, and incubated them for 16 h in LB medium supplemented with DIBAC_4_(3) and AFH633 (Fig. 4A). The vast majority of the initially nonstained cells become stained over several hours, which indicates that many cells die during the recovery phase. None of the stained cells was able to grow again. Mortality curves based on the kinetics of appearance of the stained cells showed that DIBAC_4_(3) stained cells before AFH633 and that *ΔtisB* cells were stained 1.76- and 1.59-fold faster with DIBAC_4_(3) and AFH633, respectively, than WT cells (Fig. S4). Comparison of the CFU-based survival frequencies and frequencies of staining with DIBAC_4_(3) after 20 h of TMP treatment, i.e., time zero after drug removal, indicates that around 75% and 31% of the *ΔtisB* and WT cells, respectively, die during the recovery phase after treatment. Therefore, the absence of TisB is advantageous during TMP treatment, but *ΔtisB* cells die faster during recovery phase after drug removal.

10.1128/mbio.00385-22.4 FIG S4 Post-antibiotic treatment mortality rates. Download FIG S4, PDF file, 0.4 MB. Copyright © 2022 Su et al. 2022 Su et al. https://creativecommons.org/licenses/by/4.0/ This content is distributed under the terms of the Creative Commons Attribution 4.0 International license.

### TisB modulates membrane trafficking during TMP treatment.

Many membrane transporters require energy and/or PMF and are expected to be less active in the WT cells in which TisB is induced. Because the import of thymine and thymidine depends also on such transporters (24), we hypothesized that TisB expression during TMP treatment may accelerate intracellular thymidylate depletion by blocking its import, and consequently dTTP production *via* the pyrimidine salvage pathway (Fig. S1C). In contrast, the inactivation of the *tisB* gene is expected to allow continuation of thymine import and dTTP production. Our hypothesis is supported by the observation that the inactivation of *deoR* gene, which results in an increased thymine catabolism (Fig. S1C), decreased the survival of the WT (Fig. 4B and Fig. S2C), but not of the *ΔtisB* strain.

To further confirm that TisB impacts the activity of PMF- and energy-requiring transporters, we investigated if TisB affects lactose import during TMP treatment because lactose import depends on the PMF-dependent LacY transporter (25). We added lactose to the medium after 1 h of TMP treatment and measured the induction of the P*lacZ*-GFP reporter in WT and *ΔtisB* mutant (Fig. 4C). As expected, the P*lacZ*-GFP reporter was induced significantly faster and stronger in the *ΔtisB* mutant than in the WT strain. Slopes of the linear regressions for WT and *ΔtisB* mutant were significantly different (linear regression slope comparison test, *P* value < 0.0001).

However, besides being important for uptake of nutrients, PMF may also facilitate the import of some toxic molecules, like aminoglycoside antibiotics. Therefore, we examined the impact of gentamicin (GM), whose uptake requires PMF (26), on the survival of TMP-treated cells. We first visualized the import of GM in WT and *ΔtisB* cells after 2 h of TMP treatment, by using a gentamicin-Texas Red (GTx) conjugate and microscopy imaging (Fig. 4D). We observed that GTx penetrated *ΔtisB* cells 3.4-fold more than WT cells (Mann-Whitney test, *P* < 0.0001). There was practically no difference in the fluorescence between the two strains when they were not treated (WT/*ΔtisB *= 0.9) or when they were treated only with GTx (WT/*ΔtisB *= 1.1). We also measured the survival of WT and *ΔtisB* cells when GM was added at the MIC (Table S2) after 3 h of TMP treatment. GM reduced the survival of TMP-treated *ΔtisB* mutant 161-fold more (Mann-Whitney test, *P* < 0.02) than the survival of the TMP-treated WT (Fig. 4E).

Finally, we tested if TisB can protect TMP-treated cells against colicin M (ColM). Colicins are toxic molecules produced by *Enterobacterales* to reduce competition from other bacteria in the intestine (27). Importantly, colicin-coding genes are controlled by the LexA repressor. Uptake of ColM requires its binding to the FhuA outer membrane receptor, followed by an energy-dependent translocation into the periplasm through the TonB system and the inner membrane PMF (28). Once in the periplasm, ColM hydrolyses peptidoglycan lipid precursors and causes cell lysis. We found that ColM added after 3 h of TMP treatment, reduced the survival of TMP-treated *ΔtisB* mutant 12.4-fold more (Mann-Whitney test, *P* < 0.0079) than the survival of the TMP-treated WT cells (Fig. 4F). Therefore, we showed that TisB activity could be advantageous to the SOS-induced cells when toxic molecules are present in the environment.

### Impact of TisB on E. coli’s fitness in ecologically relevant environments.

**FIG 5:**
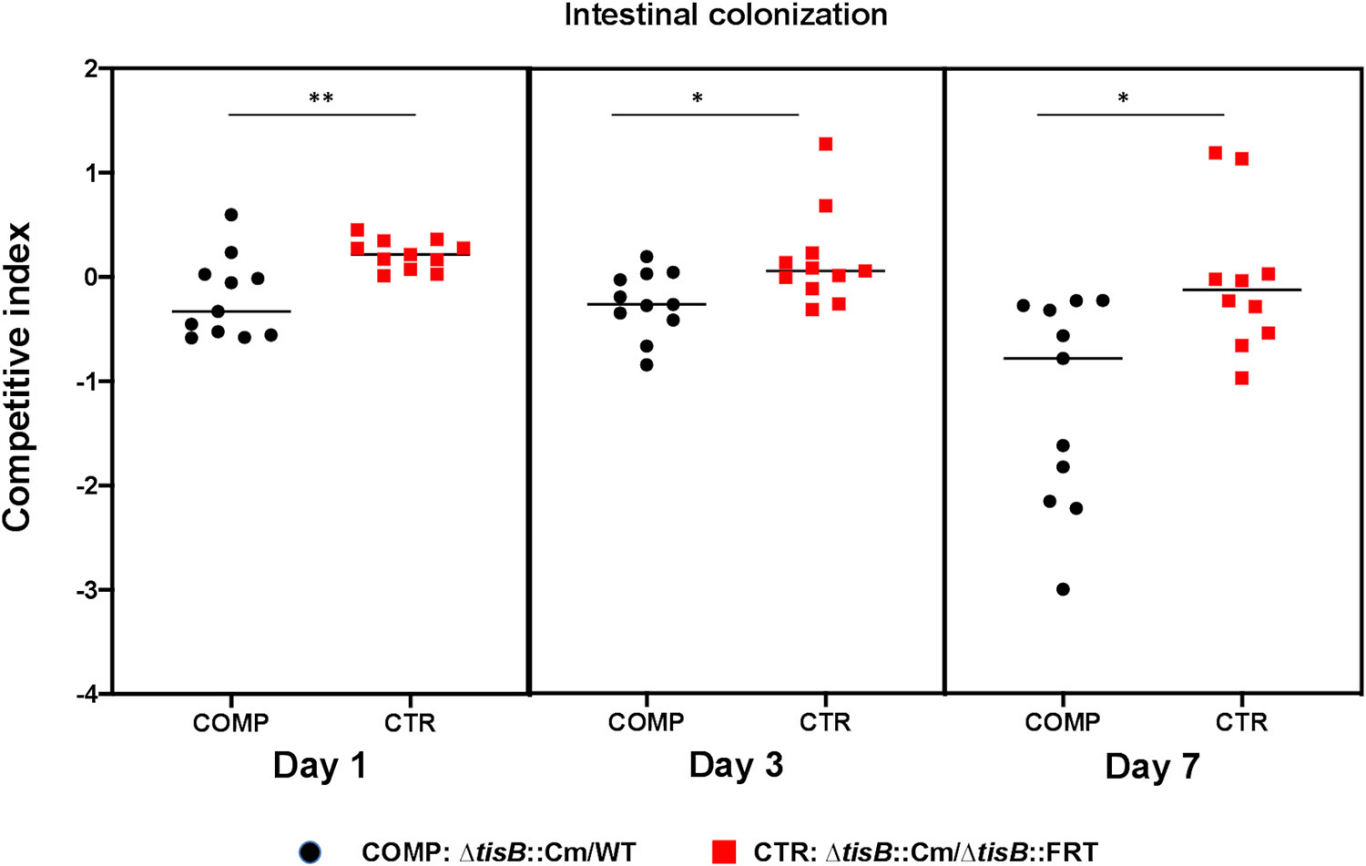
Impact of TisB on intestinal colonization. Competition between WT and the Δ*tisB* mutant during mouse intestinal colonization. Competitive indexes (CI) are given for day 1, day 3, and day 7 after bacterial inoculation. Mice were inoculated with a 1-to-1 ratio of WT and Δ*tisB*::Cm cells or a 1-to-1 ratio of Δ*tisB*::Cm and Δ*tisB*::FRT cells. The first competition (COMP) is for measuring the CI. The second competition (CTR) is to evaluate the impact of the Cm^r^ cassette on the CI. Each symbol represents the CI for one mouse. Horizontal bars represent median values. Statistical differences were calculated using Mann-Whitney test (*, *P* < 0.05; **, *P* value < 0.01).

Because *Enterobacterales* use colicins to fight competitors in the mammalian gut, which is E. coli‘s primary natural habitat, we investigated if TisB may have an impact on the colonization of mouse gut by E. coli. For this, we performed competitions between WT and *ΔtisB* strains during intestinal colonization. We used a modified streptomycin-treated mouse model, where the streptomycin treatment was stopped 5 days before the inoculation of bacteria (29, 30). The two strains were inoculated at a 1-to-1 ratio, and their relative ability to colonize the mouse intestine was determined from the fecal bacterial load at days 1, 3, and 7 postinoculation (Fig. 5). We observed a small but significant reduction in the competitive ability of *ΔtisB* mutant relative to WT after 1 day. After 7 days, competitive ability of the *ΔtisB* mutant had further decreased. The Cm^r^ cassette used for the *tisB* gene deletion has a negligible effect on the competitive index in these assays. These data suggest that TisB may contribute to E. coli’s fitness in the mammalian intestine.

### Phylogenetic analysis of the TisB/IstR TA system.

Because natural selection eliminates deleterious functions, it is plausible that the observed TisB-associated deleterious effects, like increased susceptibility to TMP due to the blocking of nutrient uptake, are largely counterbalanced by TisB-associated selective advantages in natural E. coli habitats. However, TisB may also be selected for in some ecological niches and counterselected in others. In order to answer this question, we performed phylogenetic analysis of the TisB/IstR TA system in bacteria. We found that the *tisB*/*istR* genes are present only in the Enterobacter-Escherichia clade of the order *Enterobacterales* (31), i.e., they were found in *Citrobacter*, Salmonella and Escherichia/*Shigella* strains, as well as in Enterobacter cancerogenus (formerly Enterobacter taylorae) MiY-F (Table S3). The phylogeny of the 586-bp region flanking *tisB* (90 bp) and *istR* (140 bp) follows the strain phylogeny (Fig. S5A), which was established with the multilocus sequence analysis (MLSA) using the core genome *atpD*, *gyrB*, *infB*, and *rpoB* gene sequences (31) (Fig. S5B). These observations suggest the unique, relatively recent arrival of this TA system during diversification of the Enterobacter-Escherichia clade within the order *Enterobacterales*.

10.1128/mbio.00385-22.5 FIG S5 Phylogenetic analysis of the TisB/IstR TA system. Download FIG S5, PDF file, 0.8 MB. Copyright © 2022 Su et al. 2022 Su et al. https://creativecommons.org/licenses/by/4.0/ This content is distributed under the terms of the Creative Commons Attribution 4.0 International license.

10.1128/mbio.00385-22.8 TABLE S3 Strain ID and genome accession numbers of the Enterobacter-Escherichia clade taxa possessing the TisB/IstR TA system presented in Fig. S5A and B. Download Table S3, PDF file, 0.09 MB. Copyright © 2022 Su et al. 2022 Su et al. https://creativecommons.org/licenses/by/4.0/ This content is distributed under the terms of the Creative Commons Attribution 4.0 International license.

**FIG 6:**
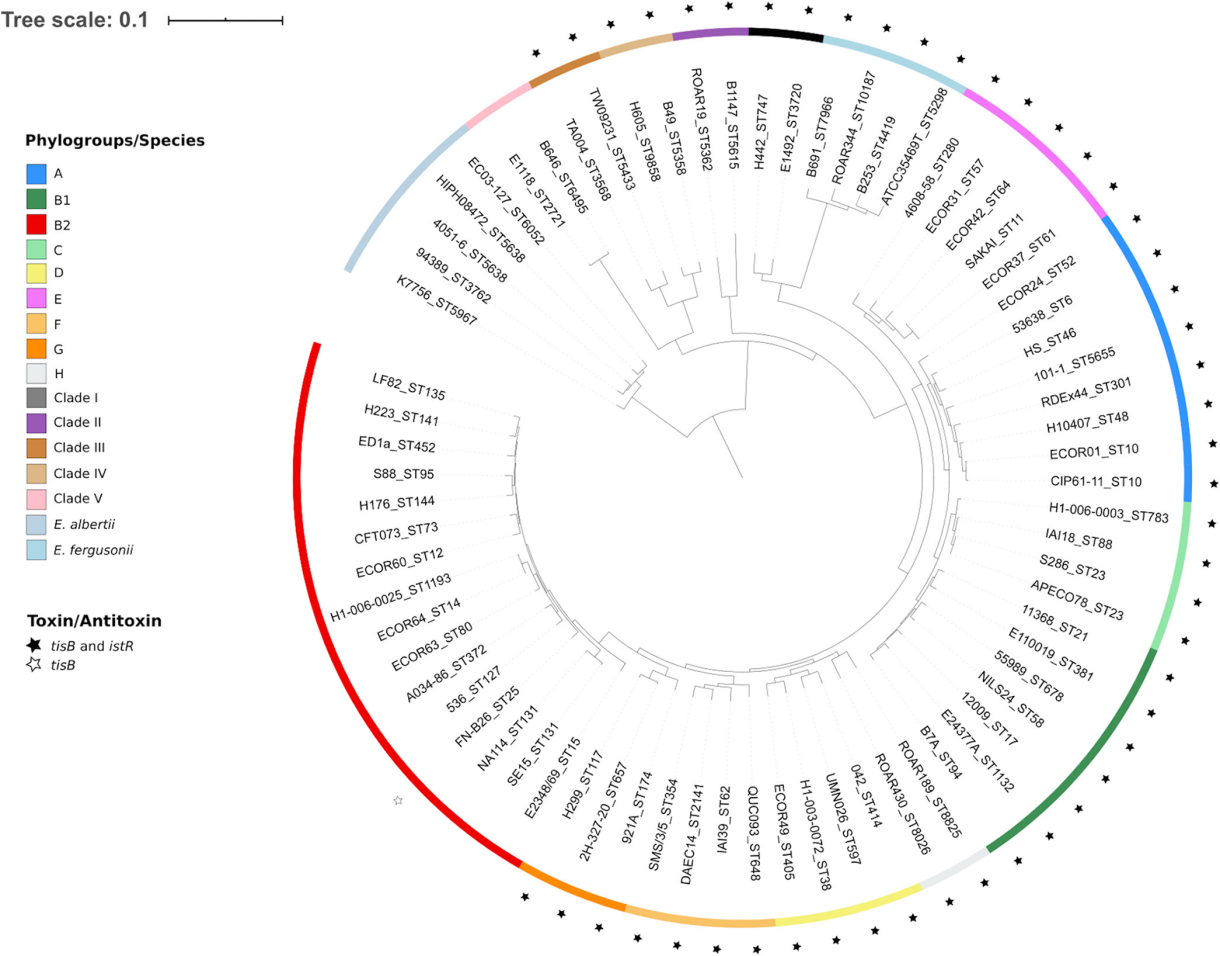
Presence of the TisB/IstR TA system in the genomes of Escherichia strains. Phylogenetic history of 72 Escherichia strains representative of the genus diversity with the presence/absence of the TisB/IstR TA system. The tree was reconstructed from the single nucleotide polymorphisms (SNPs) (*n* = 374,678) of core genome genes (*n* = 1,302) using ROARY and RAxML and rooted on *E. albertii*. The strains are identified by their ID followed by the ST number according to the Achtman (Warwick University) scheme. The external circle corresponds to the phylogenetic groups according to the given color code. The presence of the TisB/IstR TA system is indicated by a star. Of note, the B2 phylogroup E. coli FN-B26 strain possesses only the *tisB* gene, but at an unknown location in the sequenced genome.

Next, we examined the distribution of the *tisB*/*istR* genes within the genus Escherichia using a collection of 72 strains, which were chosen to represent Escherichia phylogenetic diversity (32). The analysis showed that the *tisB*/*istR* genes are present in most of the strains (Fig. 6), always at the same chromosomal location between *ilvB* and *emrD* genes (Fig. S5C), and that the *tisB*/*istR* region phylogeny (Fig. S5D) follows the phylogeny of the examined strains based on the core genome genes (Fig. 6). However, the *tisB*/*istR* genes were absent in Escherichia albertii, Escherichia clade V, and E. coli *sensu stricto* phylogroup B2 strains. Interestingly, one B2 strain, FN-B26, has an empty canonical chromosomal site of the *tisB*/*istR* gene integration (Fig. S5E), but we detected the presence of the *tisB* gene at an unknown location in the sequenced genome. Precise analysis of the *ilvB-emrD* region in *E. albertii*, Escherichia clade V, and E. coli B2 strains showed three species-specific complex deletions/insertions (Fig. S5E).

## DISCUSSION

In this study, we investigated the functional integration of the TisB/IstR TA system in the E. coli SOS genotoxic stress response regulon. This regulon, which is highly conserved in eubacteria, has a large variability of regulon members as a function of adaptation of different bacterial species to diverse ecological niches. Therefore, we investigated the presence of the *tisB*/*istR* locus in bacteria and found that it is present only in the recently emerged genera of the *Enterobacterales* order, probably due to a unique ancestral arrival event. The *tisB*/*istR* locus is present in most Escherichia clades, except in *E. albertii*, *E.* clade V, and E. coli phylogenetic group B2, each of which lost it by an independent event. The primary natural habitat of E. coli is the gut of warm-blooded animals. It was previously reported that the capacity to induce the SOS response is important for E. coli during intestinal colonization in order to compete with other members of the intestinal microbiota (33). Our study shows that TisB may be one of the LexA-controlled functions that contribute to E. coli’s fitness during intestinal colonization. We investigated which of the different TisB-induced phenotypes may provide a competitive advantage to its host.

The SOS regulon is induced by and deals with DNA damages. Therefore, we asked whether TisB participates in DNA repair or contributes to cell survival in another way. We found that the deletion of the *tisB* gene increases the survival to lethal TMP treatment. This indicates that TisB does not participate in DNA repair. However, we showed that the wild-type strain is more affected than the *ΔtisB* mutant by TMP treatment, because TisB inhibits the activity of the PMF-dependent thymine transporters and thus prevents dTTP production *via* a pyrimidine salvage pathway (15). We also showed that TisB modulates the activity of other PMF-dependent transporters, by showing that it impacts lactose import. Therefore, TisB prevents the import of nutrients like sugars. However, this is not expected to increase DNA repair capacity of the WT cells, because maintaining access to nutrients is essential for stressed cells to simultaneously deal with cellular damage and sustain a functioning metabolism (34).

However, we also showed that TisB-dependent disruption of the PMF reduces the uptake of toxic compounds, such as the aminoglycoside antibiotic gentamicin and colicin M, into the SOS-induced cells. We propose that the primary biological role of TisB toxin is to prevent the uptake of colicins (and other toxic compounds) in strongly stressed cells. This hypothesis is based on the following facts: (i) SOS induction is important for intestinal colonization because it provides a competitive edge over other microbiota members (33), (ii) colicin-coding genes are controlled by the LexA repressor, (iii) colicins are toxic molecules produced by *Enterobacterales* to reduce competition with other bacteria in the intestine (27, 51), and (iv) TisB protects stressed cells against colicin M.

Using low-copy-number-plasmid-borne transcription reporters for 12 LexA-controlled genes, we observed that *tisB* gene expression was induced by treatment with TMP at 20× MIC but not 0.1× MIC. If these plasmid-borne transcriptional reporters faithfully reproduce the kinetics of the SOS regulon induction at high TMP doses, then it can be concluded that *tisB* gene induction was later compared to other LexA-controlled genes, such as *recA* and *sulA*. This is consistent with the fact that the sequence of the *tisB* gene’s LexA box has the lowest heterology index among the LexA-controlled genes, which means that it binds to LexA with a very high affinity (35). In addition, it was shown that LexA dissociates much more slowly from the *tisB* gene operator than from other LexA-controlled gene operators (36).

A similar delay was observed for the appearance of TisB-dependent phenotypes, such as membrane depolarization or cell death upon TMP treatment. TisB-dependent membrane depolarization was also observed only after prolonged CIP treatment (37). Importantly, the colicin-coding genes are also induced only with high doses of SOS-inducing agents and with a pronounced delay (38). So, the timing of the *tisB* gene induction should allow the DNA repair machinery time to deal with DNA damages before TisB transiently blocks cell functioning in order to protect cells against colicins and other environmental toxic compounds.

The conservation of the order of induction of the LexA-controlled genes by different stressors most likely reflects the frequency and the importance with which different genes were required for dealing with recurrent genotoxic challenges E. coli experienced in its natural habitats during its evolutionary history. This capacity for predictive behavior of transcription networks based on *déjà vu* challenges is exemplified by coordinated induction of distinct transcription responses of E. coli to temperature upshift and oxygen downshift, both of which happen upon transition from the external environment to the mammalian gastrointestinal tract (39). Similarly, Saccharomyces cerevisiae responses to a succession of stresses occurring during the process of alcoholic fermentation reflect their natural temporal order of appearance in such way that the early stresses are used as predictive signals for the arrival of later stresses (40).

What would be the ecological situation that selected for the timing of *tisB* gene induction and for TisB functions? One such situation may be when bacterial pathogen-induced inflammation of the intestine induces microbiota dysbiosis. For example, it was reported that invading Salmonella enterica serovar Typhimurium induces inflammation to disrupt colonization resistance. Host innate immunity defense, such as reactive oxygen species, induces SOS and colicin production by Salmonella, which eliminate commensal E. coli strains that are major Salmonella competitors under inflammatory conditions (41). However, our study indicates that E. coli, which possesses TisB, is not disarmed against attack by colicins and other bacteriocins. Therefore, TisB toxin may provide a competitive edge to its hosts when they are simultaneously exposed to genotoxic stresses and fierce chemical warfare with other bacteria in the intestine.

## MATERIALS AND METHODS

### Bacterial strains.

Bacterial strains and plasmids used in this study are described in Table S1. All strains are derived from the MG1655 strain.

### Growth conditions.

Growth was performed in LB medium at 37°C with shaking at 150 rpm. The medium was prepared fresh by filter sterilization. If needed, antibiotics were added at the following concentrations: 30 μg/mL chloramphenicol, 100 μg/mL ampicillin, and 50 μg/mL kanamycin. For supplementation experiments, thymidine was added at a final concentration of 0.3 mM. Chemicals were purchased from Sigma-Aldrich unless otherwise indicated.

### Determination of the MIC.

The MIC of an antibiotic for the bacterial strains (Table S2) was defined as the antibiotic concentration that inhibited growth after 24 h of incubation in LB liquid medium at 37°C. Cells from fresh overnight cultures were diluted 1/10,000 (vol/vol) and inoculated into 96-well plates (Greiner Bio-One round bottom 96 well plates) containing LB medium and different concentrations of antibiotics. Microtiter plates were incubated for 24 h at 37°C in a microplate reader and incubator (Infinite M200 Pro; Tecan). The optical density (OD) at 600 nm was measured every 10 min.

### Measuring expression of the GFP-based transcription reporters.

Fresh overnight cultures were diluted (1/10,000) and inoculated in 96-well microtiter plates containing LB. For sub-MIC experiments, TMP was added to the medium at the final concentration of 0.05 μg/mL (0.1× MIC), 50 μL of mineral oil was added to each well to avoid evaporation, and the plates were incubated at 37°C in a microplate reader incubator (Spark; Tecan). For lethal conditions, the plates were first incubated in a microplate incubator at 37°C with orbital 800-rpm shaking for 4 h to reach exponential growth phase. TMP and CIP were then added to the cultures at final concentrations of 10 μg/mL (20× MIC) and 0.1 μg/mL (10 × MIC), respectively. Control cultures were incubated without treatment. OD_600_ and green fluorescent protein (GFP) fluorescence (excitation, 480 nm; emission, 510 nm) were read every 10 min for 4 h.

### Survival of bactericidal treatment.

Overnight cultures were diluted 1/1,000 in LB medium and incubated at 37°C and 150 rpm, until an early-exponential-phase OD_600_ of ∼0.2 was reached. Cultures were subsequently diluted 1/1,000 to prevent the carryover of cells from stationary phase and grown to a mid-exponential-phase OD_600_ of ∼0.6. Cells were then treated with lethal concentrations of antibiotics, either TMP at 10 μg/mL or CIP at 0.1 μg/mL. VAN was used at the sub-MIC of 0.125 mg/mL (0.25 × MIC). At given times, cells were collected by centrifugation and washed with 0.01 M MgSO_4_. Survival was quantified by plating serial dilutions of cells on LB plates before and after antibiotic treatment and by counting the number of CFU after an overnight incubation at 37°C.

### TDtest.

The TDtest allows the detection of bacterial tolerance to lethal antibiotic concentrations (42). To evaluate the impact of TisB on survival of TMP treatment, we modified the TDtest as follows. E. coli WT cells were grown in LB medium to an OD_600_ of ∼0.6, as for testing the survival of bactericidal treatments. Growth medium was sterilized by filtration. Conditioned sterilized medium was used for the preparation of plates; then, bacterial suspensions, with approximately 10^5^ bacteria/mL, were streaked using a sterile cotton swab. Absorbent paper discs were placed in the middle of the plates. TMP (50 μL; 300 μg/mL) was added on each disc. Plates were incubated 24 h at 37°C. Bacteria growing outside the inhibition zone exhaust nutrients from the whole plate (even from the inhibition zone). To allow growth of surviving bacteria, 10 μL of 30% glucose, which provides energy for growth, and 20 μL of 100 μg/mL thymidine, which counteracts the effects of TMP, were spotted on the discs, and plates were incubated 24 h at 37°C. Colonies of surviving bacteria can be visualized in the inhibition zone.

### Membrane potential assay.

Samples (500 μL) of bacterial cultures were collected and centrifuged for 2 min at 20,000 × *g*. After the removal of the supernatant, the cell pellet was resuspended in 500 μL of DIBAC_4_(3) at 2.5 μg/mL in 0.01 M MgSO_4_. After 15 min of incubation at room temperature in the dark, samples were washed in 0.01 M MgsO_4_ and then analyzed using a Gallios flow cytometer (Beckman Coulter). Single-cell fluorescence was measured using a 488-nm excitation laser and 525-nm emission filter. Cells exposed to a temperature of 90°C during 5 min were used as a positive control. A minimum of 50,000 cells were analyzed *per* experiment.

### ATP measurement.

The ATP measurement was done using an ATP determination kit (Molecular Probes). All the sample preparation steps were carried out on ice or at 4°C. At given times of the TMP treatment, 10 mL of culture was collected and centrifuged for 10 min at 4,000 rpm. The supernatant was filtered and stored at 4°C until the assay. The cell pellet was precipitated with 120 μL of ice-cold 6% perchloric acid and lysed for 10 min using lysing matrix B (MP Biomedicals). The samples were then centrifuged for 5 min at 12,000 rpm, the supernatant was collected and the pH was adjusted to ∼7 with 2 M K_2_CO_3_. A 20-μL volume of samples was mixed with 180 μL of luciferin-luciferase solution using 96-well white microtiter plates. After 30 min of incubation at room temperature, the luminescence was measured using a CLARIOstar Plus plate reader (BMG Labtech). The luminescence of ATP solutions ranging from 0.1 to 1 μM was measured to obtain a standard curve (*r*^2^ = 0.9991). The ATP concentrations were calculated using the standard curve. The results were corrected for sample biomass, approximated by the cell pellet weight.

### Determining post-antibiotic treatment mortality rates.

After lethal TMP treatment, cells were collected, washed, and loaded in a microfluidic device as described before (34). The microfluidic device we used consists of a series of growth channels through which the LB medium with DIBAC_4_(3) 2.5 μg/mL and AFH633 4 μg/mL is passed at a constant rate. The samples were monitored using a Nikon Ti-E inverted microscope coupled to MetaMorph software, and images were acquired every 20 min for 16 h. Mortality curves were analyzed using the R package growthrates (43) by applying the logistic growth model.

### PMF-dependent transporters’ activity.

WT and Δ*tisB* cells were grown in LB and treated with 20 × MIC of TMP. For cells carrying the P*lacZ*-GFP reporter, 0.2% lactose was added to the cultures after 1 h of TMP treatment and a 150-μL volume was transferred to a 96-well microtiter plates. Fifty microliters of mineral oil was added to each well. The plates were incubated at 37°C in a microplate reader incubator (Spark; Tecan), and control cultures were incubated without lactose. OD_600_ and GFP fluorescence (excitation/emission, 480/510 nm) were read every 15 min for 8 h.

To observe the import of gentamicin (GM), we used a gentamicin-Texas Red (GTx) conjugate which was obtained by mixing 4.4 mL of GM 50 mg/mL with 0.6 mL of Texas Red (Molecular Probes; 2 mg/mL) and agitated at 4°C for 48 h (44). After 2 h of TMP treatment, GTx was added to the cultures at the MIC (2.5 μg/mL) for 15 min. For control cultures, 2.5 μg/mL GTx was added to the cultures at an OD_600_ of 0.6 for 15 min. Cells were then collected and washed in 0.01 M MgSO_4_. One microliter of sample was spotted onto 1.5% agarose pads prepared with 0.01 M MgSO_4_ for microscopy imaging. For fluorescence detection, we used a 561-nm excitation laser and a 630/60-nm filter. For each observation field, we acquired 16 images by varying the Z parameter with a 0.1-μm step in order to obtain images at different focal planes and a depth of field adapted to the bacteria’s widths. Using the ImageJ Z-projection tool, the images were summed into one image for analysis. The mean fluorescence of each cell was obtained after analysis using the MicrobeJ plugin (45) on Fiji (ImageJ).

### Susceptibility of TMP-treated cells to gentamicin and colicin M treatments.

To test the susceptibility of TMP-treated cells to GM, 2 μg/mL (= MIC) of GM was added to the cell cultures after 3 h of TMP treatment. Survival was quantified by plating serial dilutions of cells on LB plates before and after the addition of GM and by counting the number of CFU after overnight incubation at 37°C.

Colicin M (ColM) was prepared using the E. coli strain C43(DE3) carrying the pMLD238 plasmid as previously described (46). Briefly, cells were grown at 37°C in 500 mL of LB medium containing kanamycin. When the OD_600_ reached 0.6, isopropyl 1-thio-β-d-galactopyranoside (IPTG) was added at a final concentration of 1 mM, and growth was continued for 3 h at 37°C. Cells were collected and washed with 100 mL of cold 20 mM Tris-HCl buffer (pH 7.4) containing 10 mM 2-mercaptoethanol, 200 mM NaCl, and 10% glycerol. The cell pellet was suspended in 10 mL of the same buffer and lysed for 10 min using lysing matrix B (MP Biomedicals). The resulting suspension was centrifuged at 4°C for 15 min at 15,000 × *g* and the supernatant stored at 20°C before purification of the His_6_-tagged protein. The purification of the His_6_-tagged proteins was performed using a HisTrap purification column. The column was equilibrated in the previously described buffer supplemented with 10 mM imidazole, and the sample was applied using a pump at a 1-mL/min flow rate. The proteins of interest were eluted with 200 mM imidazole and dialyzed overnight against 100 volumes of the buffer. The final preparation was stored at −20°C. Protein concentrations were determined by using a colorimetric assay based on the Bradford method (dye reagent concentrate; Bio-Rad protein assay). ColM (3 μg/mL; = MIC) was added to the cultures after 3 h of TMP treatment. Survival was quantified by plating serial dilutions of cells on LB plates before and after the addition of ColM and by counting the number of CFU after overnight incubation at 37°C.

### Intestinal colonization.

Six-week-old female mice (Charles River CD-1) treated with streptomycin were used to monitor the ability of the WT and Δ*tisB* strains to colonize the intestines of mammalian host as previously described (47). Ten days before inoculation, mice were isolated and streptomycin was added to the sterile drinking water at a final concentration of 5 g/L. Streptomycin was maintained until five days before inoculation. The antibiotic treatment efficacy against the coliform intestinal population was controlled by plating a pure suspension of feces in physiological water on Drigalski selective agar medium. Mice free of coliform flora were inoculated *per os* with 10^9^ bacteria in 200 μL of physiological water. Mice were inoculated with a 1-to-1 ratio of WT and Δ*tisB*::Cm cells or a 1-to-1 ratio of Δ*tisB*::Cm and Δ*tisB*::FRT as a control for the competitive cost of the Cm^r^ cassette (29). At days 1, 3, and 7 postinoculation, the intestinal population of E. coli was estimated by plating dilutions of weighted fresh feces on LB agar with or without chloramphenicol 30 μg/mL and CFU counts after an overnight incubation at 37°C. The competitive index (CI) was calculated as the ratio of chloramphenicol-resistant CFU to sensitive CFU at given times, divided by the same ratio present in the inoculum for each competition.

All animal experiments were approved by the French Ministry of Research and by the Ethical Committee for Animal Experiments, CEEA-121, Comité d’éthique Paris-Nord (APAFIS#4951-2016020515004032 v2, 2016021216251548 v4).

### Phylogenetic analyses.

*tisB* gene sequences of the Escherichia strains representative of the genus (32) were translated. We obtained 4 protein sequences that were searched in the NCBI nonredundant (nr) database using a BLASTP search to identify TisB-bearing strains from others of the same genus in the database, with identity and coverage of 90%.

*tisB* and *istR* genes as well as the *atpD*, *gyrB*, *infB*, and *rpoB* genes of the MLSA (31) were used as the gene database in Abricate for identification of these genes in the genomes (https://github.com/tseemann/abricate) with a minimum identity and coverage of 80%. Sequences were aligned with MAFFT (48, 49). Phylogenetic trees were reconstructed using the maximum-likelihood method with PhyML (50) and the Generalized Time Reversible (GTR) model.

The phylogenetic tree of the 72 Escherichia strains representative of genus diversity was reconstructed from the SNPs (*n* = 374,678) of core genome genes (*n* = 1,302) using ROARY and RAxML, as described in reference 32.

### Statistical analysis.

All analyses were done using GraphPad Prism 8 (GraphPad Software, Inc., La Jolla, CA).
