# Supplementary material for: TisB Protein Protects Escherichia coli Cells Suffering Massive DNA Damage from Environmental Toxic Compounds
Source: mBio. 2022 Apr 4;13(2):e00385-22. doi: 10.1128/mbio.00385-22 (PMC9040746; doi:10.1128/mbio.00385-22)
Supplement: FIG S1 [file mbio.00385-22-sf001.pdf]

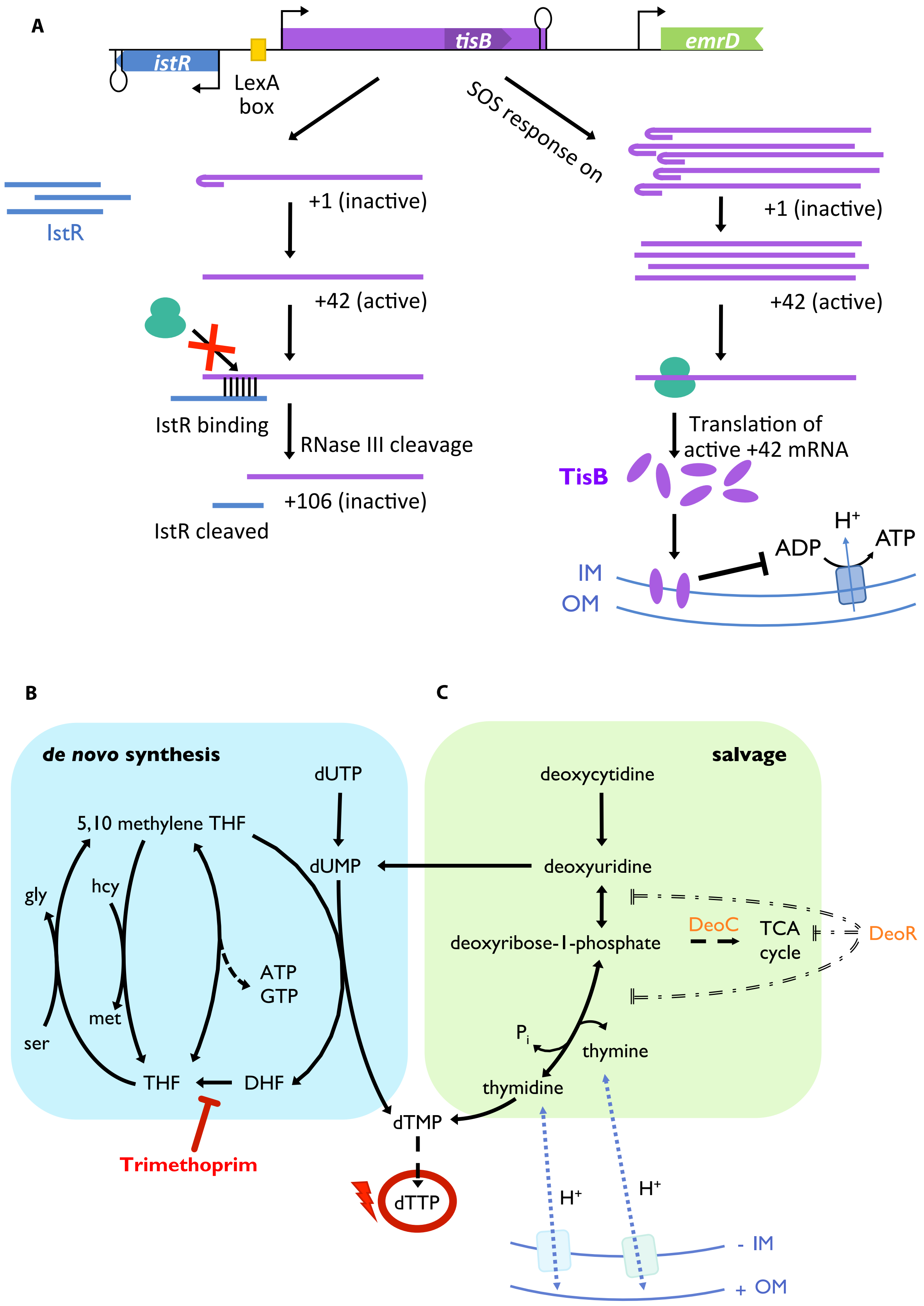
A
istR
LexA
box
tisB
emrD
SOS response on
IstR
+1 (inactive)
+42 (active)
IstR binding
RNase III cleavage
+106 (inactive)
IstR cleaved
+1 (inactive)
+42 (active)
Translation of
active +42 mRNA
TisB
H+
ADP
ATP
IM
OM
B
C
de novo synthesis
dUTP
5,10 methylene THF
dUMP
hcy
gly
ATP
GTP
met
ser
THF
DHF
Trimethoprim
dTMP
dTTP
salvage
deoxycytidine
deoxyuridine
deoxyribose-1-phosphate
DeoC
TCA
cycle
DeoR
Pi
thymine
thymidine
H+
H+
- IM
+ OM

FIG S1 Regulation of the *tisB* gene expression, folate cycle and dTTP synthesis. (A) Regulation of the *tisB* gene expression. Under normal growth conditions, SOS repressor LexA binds to the *tisB* gene LexA box and prevents its transcription, while *istR* gene is constitutively transcribed. The primary *tisB* transcript +1 is translationally inactive due to the secondary structures that prevent ribosome binding. This transcript is processed into translationally active +42 mRNA, but IstR RNA binding prevents its translation and induces its cleavage by RNase III. The cleaved *tisB* mRNA (+106) is inactive for translation. When the SOS response is induced, resulting strong *tisB* transcription overcomes IstR RNA capacity of inhibition. Consequently, TisB toxin can be synthesized. TisB protein affects the inner membrane, which results in the disruption of the proton motive force and the inhibition of the ATP synthesis. (B) Folate cycle and dTTP synthesis. TMP inhibits the reduction of dihydrofolate (DHF) to tetrahydrofolate (THF) by blocking the DHF reductase, thus blocking the folate cycle which involves the synthesis of amino-acids: methionine (met), glycine (gly); the synthesis of purines: ATP and GTP; and the neo-synthesis of deoxythymidine triphosphate (dTTP). (C) dTTP can also be synthesized through a pyrimidine salvage pathway that involves either the successive conversions of deoxycytidine into dTTP or the import of thymine and thymidine - when present in the medium - that is converted into dTTP. The salvage pathway is under the control of the DeoR repressor. DeoC, one of the salvage pathway enzymes, is responsible for the utilization of deoxyribose as a carbon and energy source.
