# Supplementary material for: TisB Protein Protects Escherichia coli Cells Suffering Massive DNA Damage from Environmental Toxic Compounds
Source: mBio. 2022 Apr 4;13(2):e00385-22. doi: 10.1128/mbio.00385-22 (PMC9040746; doi:10.1128/mbio.00385-22)
Supplement: FIG S2 [file mbio.00385-22-sf002.pdf]

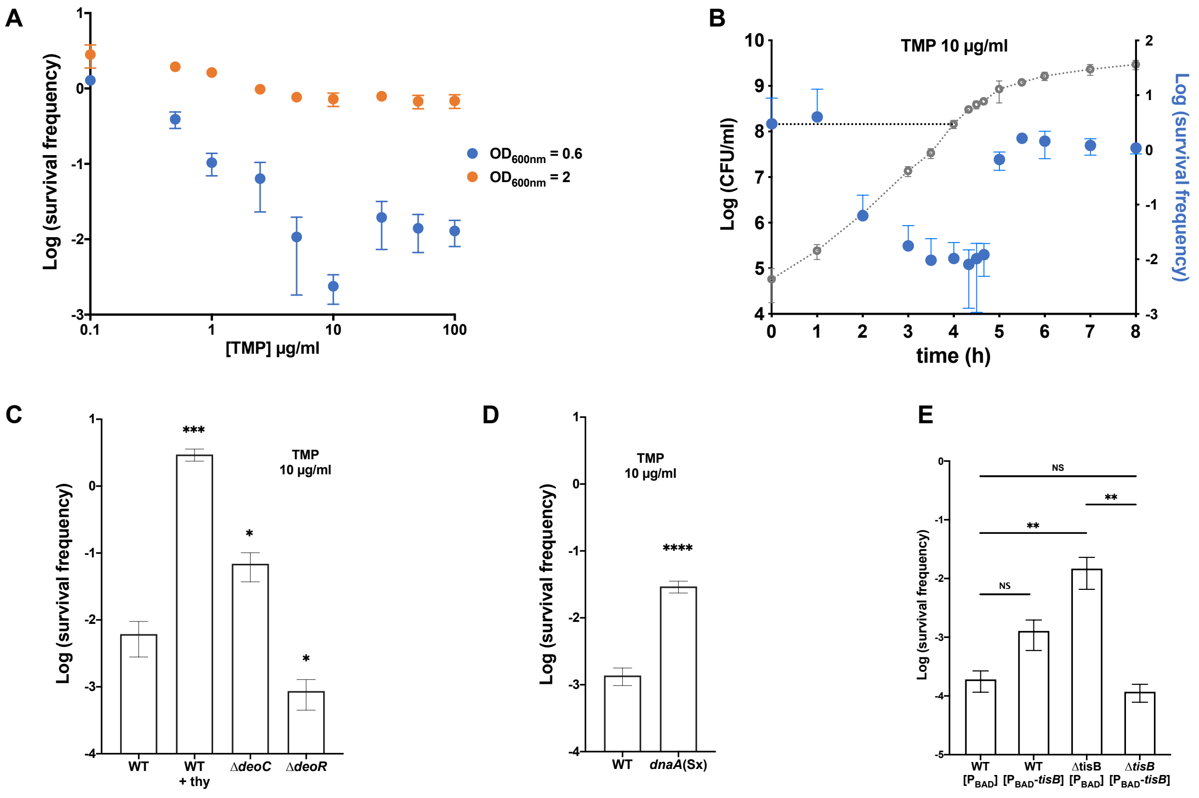

FIG S2 TMP killing activity depends on the cell culture growth phase, thymidine, the pyrimidine salvage pathway and DNA replication activity. (A) We first tested susceptibility of WT cells from different growth phases to different TMP concentrations, and decided that treatment of the exponentially growing cultures having around 2 × 10^8 colony-forming units (CFU)/ml (OD_{600nm} = 0.6) and TMP concentration of 10 µg/ml, i.e., 20 × MIC (Table 2), are most suitable for our study. This concentration of TMP was chosen because further increase of the TMP concentration did not further decrease survival, i.e., dose-response relationship displayed the Eagle effect. This cell concentration was chosen because cells at this growth phase were most susceptible to TMP. The stationary phase cells (OD_{600nm} = 2) were barely impacted. The presented results are mean values (± SEM) obtained from at least 3 independent experiments. (B) The concentrations of cells in the untreated cultures at different time points are plotted on the left axis. The dashed line indicates the density of the culture at OD_{600nm} = 0.6. At each time point, cell samples were collected and treated with TMP. Survival frequencies of treated cultures are plotted on the right axis. Mean values (± SD) of data obtained from 3 independent experiments are presented. (C) The survival of the TMP-treated WT strain in LB medium supplemented or not with 0.3 mM of thymidine, and the survival of TMP-treated Δ*deoC* and Δ*deoR* mutants. (D) The survival of the TMP-treated *dnaA*(Sx) strain, whose DNA replication initiation frequency is diminished. (E) The survival of WT and Δ*tisB* strains carrying either the P_{BAD} or the P_{BAD}-*tisB*$^+$ plasmids, to 20h of TMP treatment. Each bar represents the mean value (± SEM) of data obtained from at least 3 independent experiments. Mann-Whitney test (*vs* WT unless indicated), * p-value < 0.05, ** p-value < 0.01,*** p-value < 0.001,**** p-value < 0.0001, NS p-value > 0.05.
