# Supplementary material for: TisB Protein Protects Escherichia coli Cells Suffering Massive DNA Damage from Environmental Toxic Compounds
Source: mBio. 2022 Apr 4;13(2):e00385-22. doi: 10.1128/mbio.00385-22 (PMC9040746; doi:10.1128/mbio.00385-22)
Supplement: TABLE S1 [file mbio.00385-22-st001.pdf]

TABLE S1 Bacterial strains and plasmids used in this study

**Strain**	**Relevant genotype**	**Source**
WT	MG1655 wild-type (parental strain)	Laboratory strain collection
Δ*deoC*	Δ*deoC*::kan	MG1655 P1 (KEIO Δ*deoC*::kan, Suppl. ref. (1))
Δ*deoR*	Δ*deoR*::kan	MG1655 P1 (KEIO Δ*deoR*::kan, Suppl. ref. (1))
dnaA(Sx)	*dnaA*(Sx)721 Δ*zib*::Tn10	Ref. (15)
lexA1 (Ind$^-$)	Δ*ins*::FRT *lexA1*	Ref. (15)
Δ*sulA*	Δ*sulA::*kan	Suppl. ref. (2)
Δ*tisB*	Δ*tisB*::cm	MG1655 × *FRTcatFRT* from pKD3, Suppl. ref. (3)<br>Forward primer ATTACTGTTTATTTATACAGTAAAC<br>TTCTATAATATCACTGTGTAGGCTGGAGCTGCTTC<br>Reverse primer GTCAGCATCGCATCCGACACCAACC<br>CGCACGCTAAATATACATATGAATATCCTCCTTAG
Δ*tisB-emrD*	Δ*tisB-emrD*::cm	MG1655 × *FRTcatFRT* from pKD3, Suppl. ref. (3)<br>Forward primer ATTACTGTTTATTTATACAGTAAAC<br>TTCTATAATATCACTGTGTAGGCTGGAGCTGCTTC<br>Reverse primer TCTCCCATGGAGCTGATGACGATGC<br>TGCGGTGACGTGCGC CATATGAATATCCTCCTTAG
Δ*istR* Δ*1-41*	Δ*1-41* Δ*istR*::kan	B. Berghoff laboratory strain collection
Δ*istR* Δ*tisB*		L. Van Melderen's laboratory strain collection
Δ*tisB* Δ*deoR*	Δ*tisB*::cm Δ*deoR*::kan	Δ*tisB* P1 (KEIO Δ*deoR*::kan, Suppl. ref. (1))
**Plasmids**	**Description**	**Source**
P_{BAD}	Derived from pBAD-TOPO, amp^R	B. Berghoff laboratory strain collection
P_{BAD}-*tisB*$^+$	P_{BAD} with *tisB*$^+$ from +42 to +354	B. Berghoff laboratory strain collection
SOS genes' and<br>*lac* promoters<br>carried by pUA66	pSC101 origin, promoter region cloned<br>upstream of *gfpmut2* gene, kan^R	Suppl. ref. (4)
P_{tisB}	*istR-tisB* promoter region cloned in<br>pUA66	forward primer CTCGAGACAAAAAACCCCGCCGGAGC<br>reverse primer GGATCCACGCGTCTCCTGTGGTTCAG
pMLD238*colM*+	*colM* gene under IPTG inducible<br>promoter cloned in the pET plasmid	D. Duché laboratory strain collection

Supplementary references

1. Baba T, Ara T, Hasegawa M, Takai Y, Okumura Y, Baba M, Datsenko KA, Tomita M, Wanner BL, Mori H. 2006. Construction of *Escherichia coli* K□12 in□frame, single□gene knockout mutants: the Keio collection. Mol Syst Biol 2.
2. Dapa T, Fleurier S, Bredeche M-F, Matic I. 2017. The SOS and RpoS regulons contribute to bacterial cell robustness to genotoxic stress by synergistically regulating DNA polymerase Pol II. Genetics 206:1349–1360.
3. Datsenko KA, Wanner BL. 2000. One-step inactivation of chromosomal genes in *Escherichia coli* K-12 using PCR products.
