# Supplementary material for: TisB Protein Protects Escherichia coli Cells Suffering Massive DNA Damage from Environmental Toxic Compounds
Source: mBio. 2022 Apr 4;13(2):e00385-22. doi: 10.1128/mbio.00385-22 (PMC9040746; doi:10.1128/mbio.00385-22)
Supplement: TABLE S2 [file mbio.00385-22-st002.pdf]

TABLE S2 MIC of the studied strains for different antibiotics

**Strain**	**MIC of TMP (µg/ml)**	**MIC of VAN (mg/ml)**	**MIC of CIP (µg/ml)**	**MIC of AMP (µg/ml)**	**MIC of GM (µg/ml)**	**MIC of ColM (µg/ml)**
WT	0.5	0.5	0.01	2	2	3
dnaA(Sx)	1	ND	ND	ND	ND	ND
Δ*deoC*	0.5	ND	ND	ND	ND	ND
Δ*deoR*	0.38	ND	ND	ND	ND	ND
lexA1 (Ind$^-$)	0.38	ND	ND	ND	ND	ND
Δ*sulA*	0.75	ND	ND	ND	ND	ND
Δ*tisB*	0.5	0.5	0.01	2	2	2
Δ*istR* Δ*1-41*	0.5	ND	ND	ND	ND	ND
Δ*istR* Δ*tisB*	0,5	ND	ND	ND	ND	ND
Δ*tisB-emrD*	0.5	ND	ND	ND	ND	ND
Δ*tisB* Δ*deoR*	ND	ND	ND	ND	ND	ND

ND: not determined
