# Supplementary material for: TisB Protein Protects Escherichia coli Cells Suffering Massive DNA Damage from Environmental Toxic Compounds
Source: mBio. 2022 Apr 4;13(2):e00385-22. doi: 10.1128/mbio.00385-22 (PMC9040746; doi:10.1128/mbio.00385-22)
Supplement: FIG S3 [file mbio.00385-22-sf003.pdf]

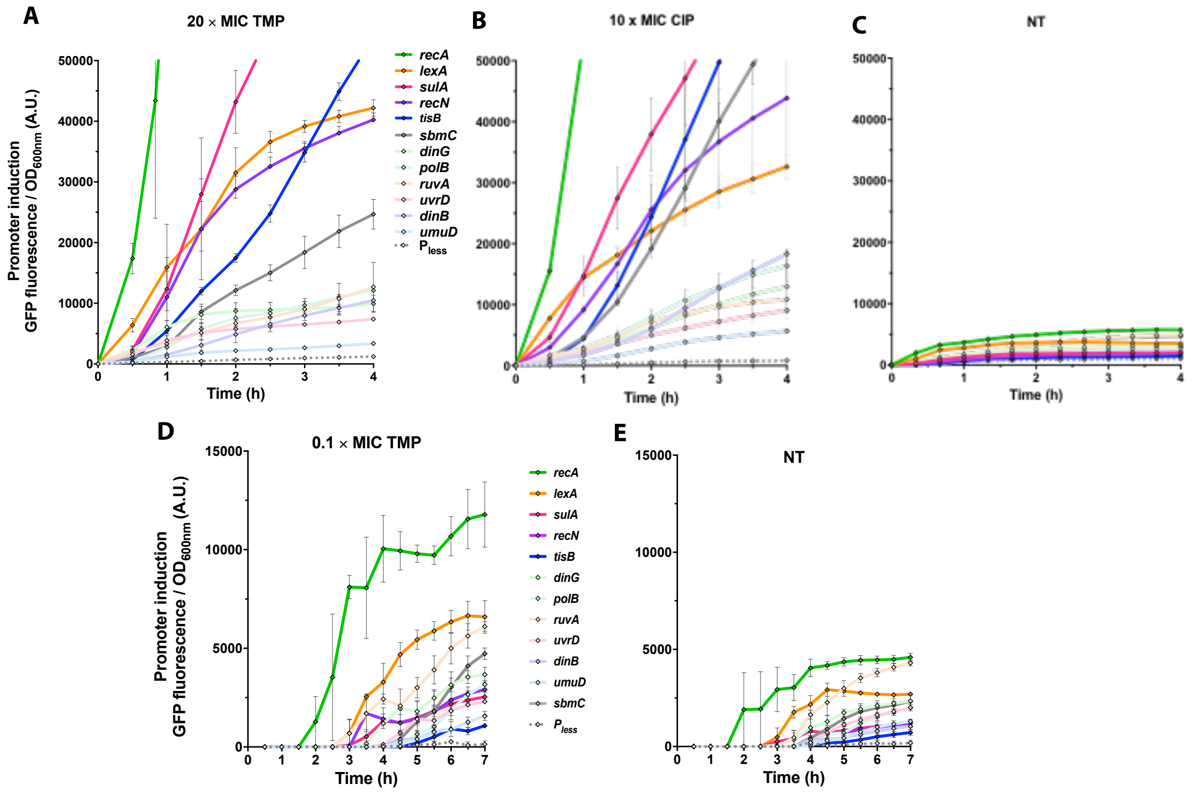

FIG S3 Kinetics of the induction of the SOS genes by antibiotic treatments. Each strain carries a plasmid with a transcriptional fusion of the *gfp* gene to the promoter of a LexA-controlled gene. Cultures of exponentially growing cells of each strain were treated with (A) 20 × MIC of TMP, (B) 10 × MIC of CIP, and (D) 0.1 × MIC of TMP. (C and E) Untreated cultures and the promoter-less (P_{less}) plasmid were used as negative controls. Each dot represents the mean value (± SD) of the results obtained from 3 independent experiments.
