# Supplementary material for: TisB Protein Protects Escherichia coli Cells Suffering Massive DNA Damage from Environmental Toxic Compounds
Source: mBio. 2022 Apr 4;13(2):e00385-22. doi: 10.1128/mbio.00385-22 (PMC9040746; doi:10.1128/mbio.00385-22)
Supplement: FIG S4 [file mbio.00385-22-sf004.pdf]

A

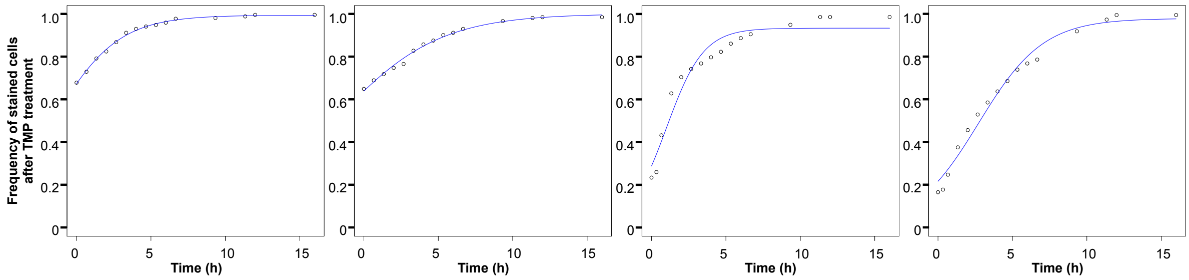

B

	Y0	μmax	K	Ratio μmax Δ*tisB* / WT
WT DIBAC$_4$(3)	0.6734724	0.4560531	0.9946111	DIBAC$_4$(3): 1.75806984
WT AFH	0.2883988	0.8017732	0.9340736	
Δ*tisB* DIBAC$_4$(3)	0.6393786	0.2888169	1.0015603	AFH: 1.59321252
Δ*tisB* AFH	0.2169101	0.4601467	0.9796009	

FIG S4. Post-antibiotic treatment mortality rates. (A) The kinetic of AFH633 and DIBAC$_4$(3) cell staining after TMP treatment is plotted as the frequency of stained cells over time. The blue line represents the fitting curve of the obtained data. The curves were analyzed using the 'growth rates' package on R and by applying the logistic growth model. (B) Three parameters obtained from the fitting: Y0, the initial death frequency, μmax interpreted as the death rate, and K as the maximum death frequency.
