# Supplementary material for: TisB Protein Protects Escherichia coli Cells Suffering Massive DNA Damage from Environmental Toxic Compounds
Source: mBio. 2022 Apr 4;13(2):e00385-22. doi: 10.1128/mbio.00385-22 (PMC9040746; doi:10.1128/mbio.00385-22)
Supplement: FIG S5 [file mbio.00385-22-sf005.pdf]

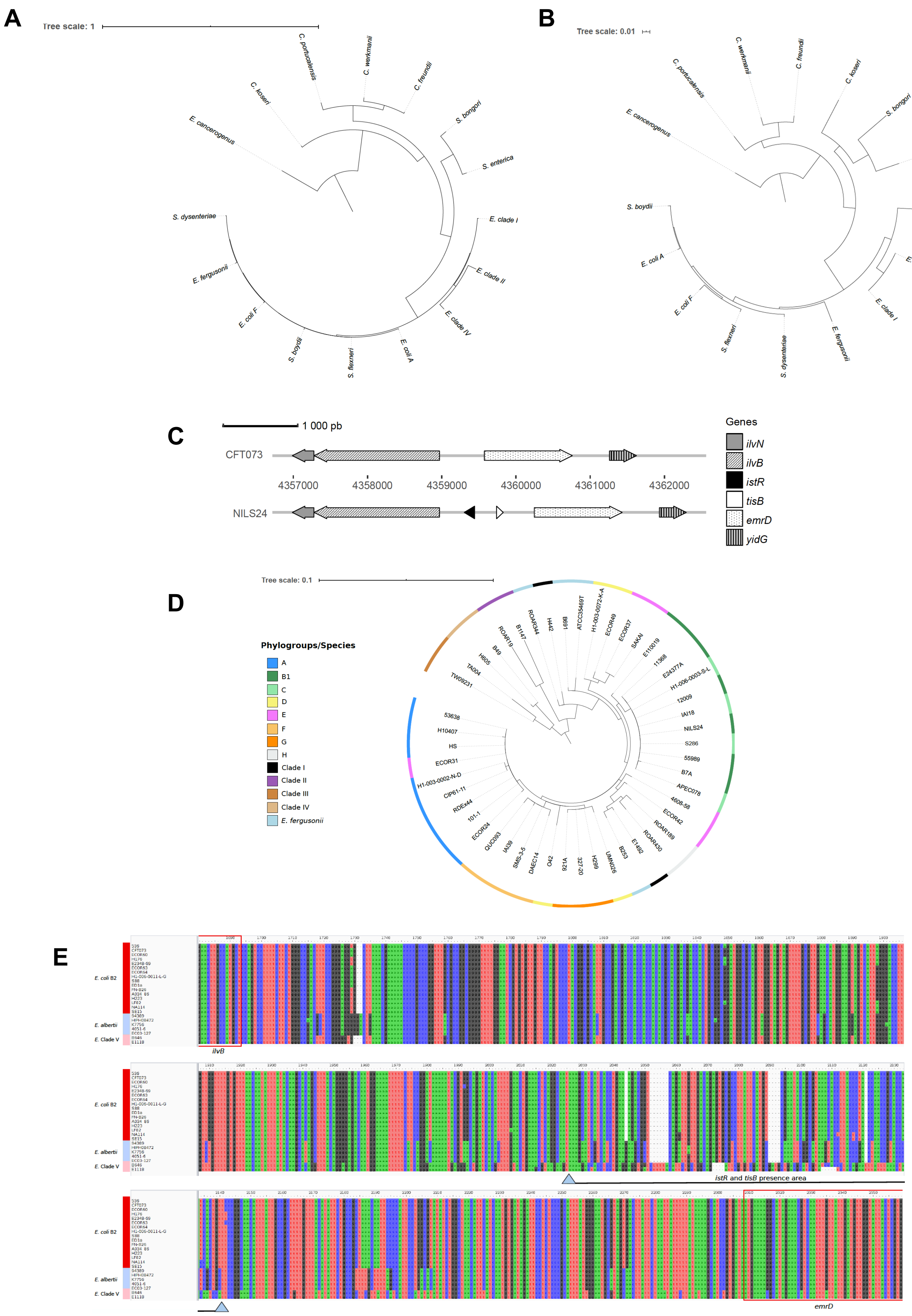
A
Tree scale: 1
C. portucalensis
C. werkmanii
C. freundii
C. koseri
S. bongori
E. cancerogenus
S. enterica
E. clade I
S. dysenteriae
E. clade II
E. fergusonii
E. clade IV
E. coli F
E. coli A
S. boydii
S. flexneri
B
Tree scale: 0.01
C. werkmanii
C. freundii
C. portucalensis
C. koseri
E. cancerogenus
S. bongori
S. enterica
S. boydii
E. clade IV
E. coli A
E. clade II
E. coli F
E. clade I
S. flexneri
S. dysenteriae
E. fergusonii
C
1 000 pb
CFT073
4357000
4358000
4359000
4360000
4361000
4362000
NILS24
Genes
ilvN
ilvB
istR
tisB
emrD
yidG
D
Tree scale: 0.1
Phylogroups/Species
A
B1
C
D
E
F
G
H
Clade I
Clade II
Clade III
Clade IV
E. fergusonii
ROAR344
H442
B691
ATCC35469T
H1-003-0072-K-A
ECOR49
ECOR37
SAKAI
E110019
11368
E24377A
H1-006-0003-S-L
12009
IAI18
NILS24
S286
55989
B7A
APEC078
4608-58
ECOR42
ROAR189
ROAR430
E1492
B253
UMN026
H299
327-20
921A
O42
DAEC14
SMS-3-5
IAI39
GUC093
ECOR24
101-1
RDEx44
CIP61-11
H1-003-0002-N-D
ECOR31
HS
H10407
53638
TW09231
TA004
H605
B49
ROAR19
B1147
E
E. coli B2
E. alberti
E. Clade V
ilvB
istR and tisB presence area
emrD

FIG S5. Phylogenetic analysis of the TisB/IstR TA system. (A and B) Maximum likelihood phylogenetic trees reconstructed from (A) the 586 bp region flanking *tisB* and *istR* and (B) the *atpD*, *gyrB*, *infB* and *rpoB* concatenated genes (2504 bp) of the *Enterobacter*-*Escherichia* clade taxa possessing the TisB/IstR TA system. The trees were rooted on *Enterobacter cancerogenus* and reconstructed using PhyML and the GTR model. The tree of the four concatenated genes corresponds to the strain phylogeny. See Table S1 for the strain ID and genome accession numbers. (C) Chromosomal map of the region surrounding the *tisB* and *istR* genes. An example of a *tisB/istR* negative (*E. coli* phylogroup B2 CFT073) and positive (*E. coli* phylogroup B1 NILS24) strains is given. The numbers below the CFT073 map correspond to the CFT073 chromosome coordinates. (D) Maximum likelihood phylogenetic tree reconstructed from the 586 bp region flanking *tisB* and *istR* of the *Escherichia* strains carrying these genes (Fig. 6). The tree was rooted on *Escherichia* clade III and IV strains and reconstructed using PhyML and the GTR model. The external circle corresponds to the phylogenetic groups according to the given color code. (E) Alignments of the nucleotides of the *ilvB* / *emrD* region in genomes of the *E. coli* phylogroup B2, *E. albertii* and *Escherichia* clade V strains lacking the TisB/IstR TA system. Grey arrows indicate positions corresponding to 5' and 3' of the *tisB/istR* region in the chromosomes of strains that possess entire *tisB/istR* TA system.
