# Supplementary material for: TisB Protein Protects Escherichia coli Cells Suffering Massive DNA Damage from Environmental Toxic Compounds
Source: mBio. 2022 Apr 4;13(2):e00385-22. doi: 10.1128/mbio.00385-22 (PMC9040746; doi:10.1128/mbio.00385-22)
Supplement: TABLE S3 [file mbio.00385-22-st003.pdf]

TABLE S3 Strain ID and genome accession numbers of the *Enterobacter*-*Escherichia* clade taxa possessing the TisB/IstR TA system presented in the Fig. S5AB.

Taxa	Strain ID	GenBank access	NCBI Reference Sequence	Assembly accession
Citrobacter freundii	CFNIH1	CP007557.1	NZ_CP007557.1	GCA_000648515.1
Citrobacter koseri	ATCC BAA-895	CP000822.1	NC_009792.1	GCA_000018045.1
Citrobacter portucalensis	Effluent 1	CP039327.1	NZ_CP039327.1	GCA_004801555.1
Citrobacter werkmanii	BF-6	-	NZ_CP019986.1	GCA_002025225.1
Enterobacter cancerogenus	MiY-F	CP045769.1	NZ_CP045769.1	GCA_009648915.1
Escherichia clade I	H442	-	-	-
Escherichia clade II	ROAR19	-	-	-
Escherichia clade IV	H605	-	-	-
Escherichia coli phylogroup A	101-1	-	-	GCA_000168095.1
Escherichia coli phylogroup F	DAEC14	-	-	-
Escherichia fergusonii	B253	-	-	GCA_000190495.1
Salmonella bongori	NCTC 12419	FR877557.1	NC_015761.1	GCA_000252995.1
Salmonella enterica	CT18	AL513382.1	NC_003198.1	GCA_000195995.1
Shigella boydii	ATCC 8700	CP026731.1	NZ_CP026731.1	GCA_002946735.1
Shigella dysenteriae	WRSd3	-	-	GCA_000499065.1
Shigella flexneri	FDAARGOS_535	CP034060.1	NZ_CP034060.1	GCA_003855135.1
